# HYRON—An Installation to Produce High Purity Hydrogen and Soft Iron Powder from Cellulose Waste

**DOI:** 10.3390/ma12091538

**Published:** 2019-05-10

**Authors:** Dan Stefanoiu, Janetta Culita, Octavian Nicolae Stanasila

**Affiliations:** 1 Faculty of Automatic Control and Computers, “Politehnica” University of Bucharest, Bucharest 060042, Romania; dan.stefanoiu@acse.pub.ro; 2 Faculty of Applied Sciences, Retiree, former member of “Politehnica” University of Bucharest, Bucharest 060042, Romania; ostanasila@hotmail.com

**Keywords:** high purity hydrogen, soft iron powder, cellulose waste, thermophysical conversion

## Abstract

The paper aims to describe a new technology of producing a gaseous mixture of type H_2_ + CO from cellulose waste that generates high purity hydrogen, without using technical oxygen. The physico-chemical process takes place in an installation designed and built for this purpose, namely HYRON, whose scheme and functionality are presented in the article, as well. The process relies on natural decomposition of cellulose waste into H_2_, CO, CO_2_ and water vapor, as a result of overheating. In subsidiary, the HYRON installation allows producing soft iron powder, with very low carbon content, from shredded iron ore, by using part of the obtained hydrogen as reducing agent. The chemical reactions underlying the gasification and redox processes, as well as the design approach of the various parts of the HYRON plant, are described in the article at length. The experimental tests made with the installation prototype (after proper calibration) showed that the resulted hydrogen had a purity of at least 99%, while the soft iron powder had over 88% pure iron in the composition. After a short economical analysis, it turned out that the costs of such products are reasonable.

## 1. Introduction

The development and the use of ecological and cost-effective technologies to produce a large amount of clean (ecological) energy constitute an imperative demand of the modern era. Over three decades, in many countries, research efforts have been made on unconventional (renewable) energies, such as wind, solar, geothermal, marine, hydroelectric and biomass-based ones. They represent an alternative to the use of fossil fuels by reducing the greenhouse gas emissions, which affect the environment. There is a worldwide continuing concern to exploit biomass, in order to employ it as a decentralized energy source.

Biomass is a versatile source of raw material, with an advanced degree of biochemical complexity, showing great potential to be a carrier of reusable substance and energy. It can be stored and transformed into gaseous fuel and electricity by specific means [1]. An important feature of biomass is that the resulted CO_2_ emissions equal the CO_2_ quantity taken from the atmosphere during photosynthesis, which represents a recirculation and not an intake from outside the atmosphere. Therefore, the term of ‘biomass’ is a substitute for recyclable organic raw materials, which can be grouped into three main categories:

- ligno-cellulosic waste (forestry or agricultural); the following are included here: tree trunks, branches, woodchips, wood pellets, bamboo, palm tree, eucalyptus [2,3], pecan nut, almond, cocoa, palm kernel-shells [4,5], sawdust waste (pine, chestnut), pine cone [6,7], wheat straw [8], stalks, sorghum, rapeseed, sugar cane, rice husk [9], corn stems and roots, Miscanthus, hemp, poplar and willow;
- algae (aquatic vegetation); subject to fermentation or anaerobic digestion, in order to produce biofuels [10,11,12];
- municipal solid waste [13,14,15]; combined with wheat straw [8], coal [16] or biomass [17,18].

Hydrogen is considered an ideal carrier of energy, since its burning has no harmful effects on the environment (even though its storage requires more attention, being easily explosive). Moreover, the methods of hydrogen production from biomass are promising from ecological perspective and economically sustainable. A comparison of such methods, extended to different renewable energy sources (including biomass), can be found in [1]. Hydrogen can be obtained from synthesis gas (mainly, H_2_ and CO), and then separated, in order to produce high purity hydrogen (h-p-H_2_) [12]. In this aim, various conversion procedures of biomass, possibly co-fed with methane [19] or other additives, are applied. These procedures are either thermochemical (pyrolysis, gasification) [20] or biochemical (anaerobic digestion, fermentation) [21]. Currently, the gasification process uses air, steam [4,22], air-steam [6,19,23], air-oxygen [24], steam-reforming [25] and supercritical water medium [2,12,25]. In [26], a state-of-the-art regarding biomass gasification technologies is presented. There are two main gasification methods. The first one is fluidized-bed gasification [6] or dual-bed gasification [27], at a temperature of 900 °C (eventually, with catalytic reforming or by using catalysts, such as: Fe_2_O_3_/CeO_2_ [28], potassium salt (K_2_CO_3_) [29], CaO [30], Ni-Cu/γ-Al_2_O_3_ [14], NiO [31]). The second one is entrained flow gasification, at about 1300 °C, and requires preheating [32]. 

In some thermochemical processes, hydrogen is produced in iron (hematite) and steam based chemical looping process [33,34]. In [22], the iron oxide is firstly reduced with carbon oxide and/or hydrogen (or mixture of both), being subsequently reformed in a reverse reaction with steam, which finally results in pure hydrogen production. In addition, a number of patents address the issue of generating hydrogen from biomass and iron oxides. For example, one patent [35] addresses the method of producing hydrogen from overheated steam, which is transferred through a room with two opposite walls: one made of iron (which reacts exothermically with the overheated steam, with hydrogen release and iron oxidation) and the other one consisting of a palladium membrane, permeable to hydrogen, but not to steam. The main disadvantages of this method are the difficulty of achieving a continuous process, as well as the low productivity, along with the high costs of the exploited palladium. Another patent [36] proposes a method of hydrogen production through the “steam-iron” reaction, by using the alternation between oxidation and reduction, at temperatures below 700 °C (actually, steam oxidation generates hydrogen). The main disadvantage is that the process is discontinuous, with agent alternations that complicate the technology of any installation and do not avoid iron carburization. A third patent [37] refers to a method and an installation of producing hydrogen through steam reduction, by using a couple (metal, metal oxide) to extract oxygen from water. The steam encounters a mixed metallic melting that includes a primary reactive metal like iron (Fe), dissolved in a diluent metal like tin (Sn). The reactive metal oxidizes, generating hydrogen. The main drawbacks of the process are, on one hand, the reduced specific surface of the varnish in the melting, which does not allow industrial applications, and, on the other hand, the use of technical oxygen, the formation of slags and heating at temperatures above 1200 °C; all these issues increase the price of proposed technology.

This paper describes a new technology to produce gaseous mixture of H_2_ + CO type, referred to as synthesis gas (syngas). This syngas is generated in continuous flow, under pressures up to 10 bar, from abundant and renewable raw material (unpelletized iron ore (e.g., hematite), water and sawdust, active charcoal or cellulosic waste). The h-p-H_2_ is then extracted from syngas. The quality of the obtained h-p-H_2_ is equivalent to the one obtained from water electrolysis, but its price is lower. The described procedure overcomes many drawbacks outlined before. For example, the gasification of the raw material is carried out by alternating flow, so that, as it descends into the corresponding gas reactor, it reacts with the gasifier agents (steam or oxygen), against their flow, by passing through various stages, until obtaining the syngas [38]. This gasification process is crucial for the high efficiency of the achieved installation, introducing a new approach. As a significant application, the resulting hydrogen can be used as reducing agent of iron ore, with the aim of simultaneously obtaining soft iron powder (s-Fe-p) with very low carbon content on one hand, and new h-p-H_2_ on the other. To the best of our knowledge, there are no reported results in the literature concerning simultaneous production of h-p-H_2_ and s-Fe-p. The generation of s-Fe-p is performed with simple, inexpensive and technically efficient means, which allow iron oxide complete reduction at a temperature of maximum 600 °C, through a loop of successive redox reactions. Thus, partial deoxidation of ore (with s-Fe-p forming) and steam oxidizing of iron (with oxide reforming) occurs first. Then, the h-p-H_2_ is produced and re-entered (together with some other h-p-H_2_ coming from the gas reactor) as reduction agent. The reoxidated ore is recirculated several times, having only the role of exchange agent in the redox reactions.

Regarding the s-Fe-p production, several procedures or technologies are known. The patent [39] introduces a method for reducing the iron ore by means of preheated hydrogen, which could contain up to 20% other gases, such as CO, N_2_ and CH_4_. The gas mixture flows against the ore, which gravitationally descends through three types of reduction rooms: a preheating one, at low temperature (300–400 °C), an intermediate heating one at medium temperature (450–600 °C) and a final heating one at high temperature (700–900 °C). The released heat is partially recovered in a special precinct, where the reducing gas is reheated. This method is one of the first to deal with iron ore reduction, the overall efficiency being quite low. Another method is based on the H-IRON technology, used in the Western European and North American industries. This technology is applied at high pressure of over 30 bar, in h-p-H_2_ atmosphere, which is not only consumes the most energy (18 GJ/t Fe), but is also in high demand of technical oxygen. In an alternative technology, known as ‘Höganäs’ (after the Swedish concern with the same name—the manufacturer of many types of metallic powders), one employs melted, pulverized and cooled scrap iron, forming a crude powder, annealed in hydrogen atmosphere, which is then chopped and grinded in a fine iron product. The disadvantage is that accumulations of powder on flat surfaces have been reported and accidents occurred, due to the spontaneous powder ignition (as the powder is flammable and difficult to control). Another known solution, newer than the mentioned ones, belongs to a patent [40] that proposes a technology of first producing an iron sponge and then reduced iron powder. Here, the iron oxide and the solid reducing agent are chopped and alternately stored in a spiral. The main drawbacks are low efficiency of reducing gas use and (again) the need for technical oxygen. 

The procedure proposed in this article mitigates the disadvantages of other solutions. Accordingly, in a first stage, the relatively dry chopped iron ore is grinded (or acquired already grinded from a steelmaker) to a granulation of 20 to 250 μm, without pelletizing. This removes the final grinding of the obtained soft iron, which otherwise would have the shape of flat blades rather than small grains. Another advantage of the proposed technology is that the plant that applies this method is compact, takes a small surface and can be built from native materials, without high investment and exploitation costs. 

There is a motivation in choosing this application of hydrogen utilization. The steel industry is in a continuous process of technological reorganization, taking into account the asset requirements, the market fluctuations, the raw material shortage (coke, ore, pure scrap iron) and, just as importantly, the requirements of modern steelmakers. DRI (Direct Reduced Iron) or HBI (Hot Briquetted Iron) technologies [41] show the trend to remove both the agglomeration processes and the necessity of coking coal, by fulfilling the goal to recycle various metallic scraps, as well. Recently, the iron scraps (which stand for the main raw material for electrical steelmakers) have been increasingly altered with harmful chemical elements and organic compounds, which requires expensive pre-cleaning and pre-sorting. Therefore, the steelmakers are continuously interested in pure iron (i.e., without carbon, phosphorus, silicon, tin, lead and others). This article offers a solution in this aim. The use of hydrogen as a reducing agent provides pure soft iron, essentially carbon-free, in the form of fine powder. 

The article is structured as follows. Section 2 describes the chemical reactions underlying the proposed method and technology. A detailed description of the installation that allows the implementation of technology (referred to as HYRON), along with the operating mode also are provided. The design of HYRON installation, with numerical calculations regarding its main components, is developed within this section. Section 3 describes the operating mode of the experimental plant that has been built (based on the design in previous section) and the results obtained during the exploitation. Finally, Section 4 concludes the article and foresees some future developments. 

## 2. Materials and method

### 2.1. Basic Chemical Reactions

As mentioned in Introduction, the article describes a new method for continuously producing of two chemicals: high purity hydrogen (h-p-H_2_) (obtained from a gaseous mixture of H_2_ + CO type, using abundant, renewable raw material (basically, cellulose waste) and soft iron powder (s-Fe-p), with low-carbon content (mainly obtained by using a part of the previously produced hydrogen). The two products are obtained in an aggregate consisting of a gas reactor (GR) and an iron reactor (IR), which are interconnected and assembled into an experimental installation (as described in Section 2.2). 

The main reaction inside the GR is the following:

2C + 2H_2_O + O_2_ + Heat → CO + CO_2_ + H_2_O^vapor^ + H_2_. (1)

The raw material is the cellulose waste with some humidity (e.g., wet sawdust (wsd)). When heating the cellulose waste up to 1000 °C (through a furnace), the water starts to vaporize generating steam, while the main cellulose material turns into carbon. The carbon immediately combines with the oxygen both from steam and atmosphere, mainly leading to CO and CO_2_ (note that O_2_ from the left side of reaction (1) is not technical oxygen). As the carbon has taken the steam’s oxygen, the hydrogen stands alone. Thus, a mixture of CO, CO_2_, H_2_ and H_2_O^vapor^ (actually, the syngas) outputs the GR at high temperature (about 1000 °C). From the hot syngas, the water vapor is condensed by cooling. This process cleans the syngas from some CO_2_, as: H_2_O + CO_2_ → H_2_CO_3_ (carbonic acid, actually a water soda). Furthermore, if necessary, the CO_2_ can be extracted from soda by decarbonizing and then stored for industrial use (although its participation in syngas is rather small). The cleaned syngas, containing H_2_ and CO, still keeps some amount of CO_2_. From the syngas, H_2_ is extracted by absorption and cooling, while CO+CO_2_ is separated by desorption and sent back to GR (actually, to the furnace). Some of the cold H_2_ is stored for industrial use. Some other cold H_2_ is reheated (by the hot syngas produced into the GR) and sent to the IR as reducing agent. The chemical reaction (as expressed in Equation (1)) was used as the basis for the calculation of this work to simplify the more typical formula used for biomass gasification reaction (involving C_x_H_y_O_z_ as the main reactant).

Within the IR, hydrogen is produced as well, although the main outcome is the iron powder, through redox reactions. This process uses unpelletized iron ore as raw material, more specifically hematite (FeO + Fe_2_O_3_), homogeneously mixed with a solid fuel of cellulosic waste or charcoal type, having the same granularity as the ore. In order to avoid segregation of adjacent iron grains and mass formation, an inactive chopped material such as mono-granular quartz sand is introduced into the IR. The resulted mixture, consisting of ore, solid fuel and sand, is heated (and thus dried) by usual thermal means (generally, with steam generated from the process, recirculated and reheated, which actually represents the recovered heat). Afterwards, it is subjected to an advanced carbon deoxidation stage at no more than 600 °C. One of the main reactions to reduce iron ore is due to carbon. More specifically:

FeO_x_ + C → Fe + αCO + (1 − α)CO_2_, (2)

where, from one batch to another, the stoichiometric coefficient α (of carbon monoxide) can vary. Through this reaction, a gas mixture of CO and CO_2_ is generated. 

After the deoxidation zone, with a reduction of at least 90%, a small quantity of preheated steam, with alternate circulation, is introduced from outside in the solid mixture, formed from the raw material (ore), sand and solid fuel (cellulose waste or coal). It gasifies the rest of the carbon, reoxidizing the previously produced iron (in Equation (2)), at a very low efficiency, thus reforming the iron ore. Simultaneously, H_2_ is released. Thus, through the reaction:

Fe + H_2_O^vapor^ → H_2_↑+ oxides (FeO), (3)

the granular mixture oxidates by producing h-p-H_2_ (over 99%), with a similar purity as the one obtained by water electrolysis, while reforming the ore. 

The alternation of the steam flow ensures the uniformity and intensifies the oxidation process. (The mathematical model of alternation and the implementation approach are described in [38].) After oxidation, the granular mixture consisting of about 90% Fe and 10% oxides (actually, the s-Fe-p) is cooled and collected. The reformed and extracted ore is homogeneously mixed with another batch of cellulose waste or charcoal, re-inputting the IR. This cycle repeats several times, until the ore is exhausted. 

The hydrogen produced by the IR (together with some hydrogen coming from the GR) is entirely employed to obtain s-Fe-p. The general reaction of hematite reduction by hydrogen is the following:

Fe_2_O_3_ + 3H_2_ = 2Fe + 3H_2_O^vapor^. (4)

However, in order to speed up the iron ore deoxidation, the reaction (4) is replaced by the following:

Fe_2_O_3_ + 15H_2_ + 12H_2_O^vapor^ = 2Fe + 15H_2_O^vapor^, (5)

which increases the amount of necessary hydrogen as reduction agent. 

In order to obtain high efficiency, the deoxidation process occurs at high temperature (but no more than 600 °C). Therefore, the iron ore has to be preheated and crossed by a flow of hydrogen with the same temperature. Hydrogen reacts with oxygen from the iron oxides to partially produce H_2_O^vapor^. The resulted gaseous mixture H_2_ + H_2_O^vapor^ is cooled in two stages, from the pre-heating temperature to a much lower temperature. The condensed water and the dust (collected from the ore) are purged and the dried H_2_ is separated for recirculation inside the IR. Thus, the hydrogen flow consists of one fresh part (supplied by GR) and one recirculated part in IR. (A more detailed description of h-p-H_2_ and s-Fe-p production process can be found in Section 3.)

After complete deoxidation, the mixture of s-Fe-p and sand is heated at high temperature, in order to remove the pyrophoricity of iron, and then, it is suddenly cooled. Next, the cooled powder, drained from the IR, is collected in a sealed metallic box, connected to a vacuum pump, in order to remove the residual hydrogen from the powder mass. To prevent re-oxidation, the sealed box is supplied with an inlet flow of argon, which easily penetrates through the s-Fe-p pores and into the intergranular space, thanks to its monoatomic structure. After a few minutes of relaxation, the s-Fe-p with reduced pyrophoricity can be loaded into bags and sent to the user. The sand is mixed and homogenized with fresh iron ore, which supplies the IR as a new batch.

### 2.2. HYRON Installation Description

**Figure 1:**
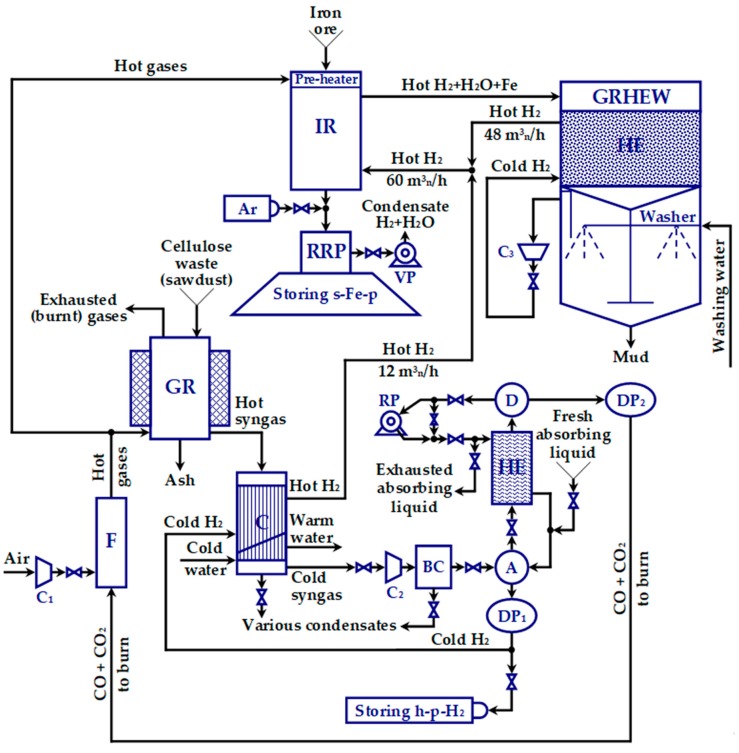
Scheme of HYRON installation.

According to Figure 1, the installation that allows implementation of the proposed method is rather complex and includes many blocks. Starting from the words hydrogen and iron, the name of this installation was ad hoc set to HYRON. From a technological perspective, its blocks have been designed appropriately, in order to achieve the desired production capacity. They are described next. 

#### 2.2.1. The Furnace (F)

The furnace (F) generates the heat and initiates the cellulose waste gasification. The compressor C_1_ operates as a blower at pressure of 1.1–1.2 bar and has a technological volumetric flow of approximately 50 m^3^_n_/h air. Recall that, for any gas, 1 m^3^_n_ is its volume at 0 °C. In the physical installation, this compressor can work with volumetric flows up to 600 m^3^_n_/h and pressures up to 8 bar. The combustion process takes place in a discharge room, where the combustible gas is combined with the compressed air. 

This room includes a whirling gas mixing system, which is designed to increase the efficiency of the heat transfer. It is lined with two thermal insulation layers (foils): the first one is of aluminium type and the second one is of basaltic type. The room was built of refractory steel, as the inner temperatures rise up to 1100 °C.

#### 2.2.2. The Gas Reactor (GR)

The gas reactor (GR) is one of the key components of HYRON plant and contains innovative constructive elements. It performs both drying and gasification of the cellulosic waste, in order to produce hydrogen. The main raw material for hydrogen production is the sawdust, with an initial humidity of approximately 35%. To produce 15 m^3^_n_ H_2_/h of high purity, one consumes 19 kg/h of wsd (i.e., with approximately 35% water), which is equivalent to approximately 14 kg/h of dry sawdust (dsd). (Other cellulose wastes can also be employed.)

The GR consists of an assembly of coaxial vertical cylinders and an external isolating cylinder (as suggested in Figure 1). The inner cylinder group is conceived so that it creates spaces for vertical ascending flow of the heater gaseous agent, alternating with the spaces for sawdust descending flow. For the systematic rummage of biomass, the middle cylinder rotates between the heated surfaces designed to give the necessary heat for the physico-chemical evolution of the wsd. This cylinder is equipped with radially shaped profiled blades. The outer cylinder is fixed and has a set of heated (hot) radial counter-blades, in order to increase the efficiency of the heat exchanged with wsd. 

To avoid dilution of useful gases with combustion gases, by direct mixing of combustion gases with processing raw material, intensification of recuperative heat exchange is needed. (For dsd, when applying other current solutions, different from those described here, the global heat transfer coefficient would result in values below 10 kcal/m^2^/h/K.) In this aim, the example of the convective heat exchange has been extended from fluids to sawdust. By using the ribbed blades system, placed both on the mobile and the fixed cylinder, the sawdust near the heating marginal surfaces is continuously mixed with the colder one that feeds the GR. A quasi-stationary homogenization of sawdust is thus realized. The heat transfer is realized both by conduction (as in the case of solid thermal agents), through the ribbed blades (which gives the heat to the moving sawdust) and convection, by transporting the warmer mass of the sawdust to the colder areas and conversely.

The blades row at the top of GR has the role of pushing the wsd from the feeding bunker, so that it descends through the whole gasification space. The upper pressing of the wsd also ensures a sealing at the top of GR, by blocking the output of ascending gases and by forcing them to descend back through the GR body. Normally, the GR operates at the atmospheric pressure (1 bar). However, because of slight upper sealing, the pressure may increase up to 1.1 bar inside the GR. In this way, pyrolysis of the generated gaseous mixture occurs and the resulted syngas includes H_2_, CO, CO_2_ and H_2_O^vapor^. The following blade rows are in charge with the homogenizing of the descending sawdust, thus ensuring its drying, pyrolysis and gasification, in a continuous process. In this manner, one can ensure that the sawdust close to the hot wall is downwardly pushed and forwarded towards the central colder zone. This movement of the sawdust mostly contributes to an efficient heat transfer. From the initial sawdust, the gasification process also yields two residuals: the ash and some burnt gases, which have to be evacuated at the GR bottom and top, respectively. The syngas is sent outside GR to the next block, the cooler (C). 

#### 2.2.3. The Cooler (C)

The cooler (C) is an important component of the installation, where the heat transfer of syngas, coming out of GR (a mixture of H_2_, CO, CO_2_, H_2_O^vapor^) takes place in two stages. The first stage occurs in the upper part of the cooler, where the syngas with the volumetric flow of about 28.7 m^3^_n_/h, enters the cooler at a temperature of about 1000 °C and leaves it at a temperature of about 300 °C. The syngas transfers the heat to a flow of 12 m^3^_n_/h H_2_, which enters the C bottom at a temperature of 20 °C and exits the C top at a temperature of approximately 600 °C. In the lower part of C, the second stage occurs, where the syngas is cooled from 300 °C to 25 °C. This heat transfer is realized by means of cold water entering the C at initial temperature of 20 °C. The cooling water outputs the C with a temperature of 35 °C at least, for a flow of about 0.7 m^3^_n_/h, and of 98 °C at most, for a flow of 0.07 m^3^_n_/h. In this stage, the condensation of water vapor from syngas is produced while it is being mixed with some CO_2_. The resulted soda water is purged through a drain pipe (with valve) at C bottom. The experiments showed that the syngas outputs the GR at about 1000 °C, with the following composition: 51.3% H_2_; 44.1% CO; 2.1% CO_2_; 2.5% H_2_O^vapor^. After cooling at 20–25 °C, the following composition is estimated: 52.26% H_2_; 44.9% CO; 1.44% CO_2_; 1.4% H_2_O (condensate).

The cold syngas is then sent to the compressor C_2_, which slightly raises the temperature to approximately 30 °C, by increasing the pressure from 1–1.1 bar to 2 bar, for a flow of approximately 14.8 m^3^/h. From C_2_, the syngas enters the next block, A-HE-D (described in Section 2.2.4), where the mixture of CO and CO_2_ is separated from H_2_. This process generates h-p-H_2_ with an estimated purity of 95–98%. Part of h-p-H_2_ is stored for subsequent exploitations as an alternative source of clean energy. The remaining h-p-H_2_ is feeding back the C, where it will play a dual role: as cooling agent for the syngas supplied by GR and as reducing agent (after being heated to about 950 °C) for the iron ore within the IR.

#### 2.2.4. The Group Absorber-Heat Exchanger-Desorber (A-HE-D)

The group Absorber-Heat Exchanger-Desorber (A-HE-D) aims to perform the gases separation in the syngas coming from the cooler. In context of HYRON installation, separation is mostly concerned with hydrogen extraction from syngas based on a selective absorption process of CO and CO_2_. Nowadays, separation of varius compounds from a mix can be realized by various methods [42]. In general, separation occurs through a cascade of processes or a process that repeats in a loop. Such processes are concerned with distillation, absorption, adsorption and membranes use. In case of gases separation from syngas, two methods are of interest: one that relies on absorption [43] and another one that employs membranes [44]. The first method involves using some absorbent liquid (e.g., copper-ammonia solution), which has to be inserted from outside the syngas room (via a funnel) and recirculated in a continuous absorption-desorption process until exhausted. This process can be exothermic. In the second method, membranes are employed to perform separation of various gases such as nitrogen, oxygen, helium, argon, carbon dioxide and even hydrogen. This time, the process occurs at constant temperature. The membrane is made of polymeric, organic, inorganic and hybrid materials. Polymeric membranes are widely used, since they have quite a low cost, are easy to install and their efficiency is satisfactory. However, extracting hydrogen from syngas, while preserving its purity, is a highly demanding task that requires quality and quite expensive membranes. Therefore, the first method was preferred in case of HYRON installation. 

The absorption of gases in liquids is a slightly exothermic process, while the desorption is a slightly endothermic one [43]. The syngas pressure is of about 1.1 bar before entering the compressor C_2_, and rises to about 2 bar when exiting the compressor. The compressor C_2_ is especially designed for medium pressures (up to 15 bar) and small flows (below 600 m^3^/h). The syngas compression process is highly exothermic (the temperature can rise from 10 °C to over 100 °C). To compensate the increase of temperature, a buffer cylinder (BC) of 5–10 l capacity is added at compressor exit. It has three pipes: one for the compressed gas inlet; one for the compressed gas outlet entering then the absorber (A); one to eliminate any condensation, compressor fluids, etc.

The syngas enters the lower zone of A, through a distributor and is initially sparged against the flow of absorbent liquid. The extracted h-p-H_2_ is evacuated and then enters the depressurizer DP_1_. The depressurizer lowers the gas pressure to 1.2 bar and sends the h-p-H_2_ in two directions: towards C, as cooling agent, and towards storing, as an alternative source of clean energy. The A contains a drop deflector, which is designed to capture the mist formed by the hydrogen bubbles with the introduced absorbent liquid. The captured absorbent fluid is directed to the inlet zone, and hydrogen is drained with minimal residuals of moisture.

From A, the absorbent liquid (saturated with CO + CO_2_) is first captured and discharged, being afterwards introduced into the heat exchanger (HE) (built with pipes). This has the role to maintain the following operating temperatures: 25–30 °C for A, 55–60 °C for the desorber (D). The absorbent liquid rich in CO flows through the pipes of HE, having the input temperature of 30 °C and the output temperature of 60 °C. The gas-free absorbent liquid flows between the pipes of HE, having the input temperature of 55 °C and the output temperature of 25 °C. The absorbent liquid, saturated with heated CO + CO_2_, is introduced in the upper zone of D, through a nozzle distributor. The working pressure in the whole A-HE-D modular assembly is of 2 bar. The absorbent liquid is captured and evacuated from D, then re-enters HE by means of a recycling pump (RP), in order to be cooled. From there, the absorbent liquid feeds A, at the upper level. After exiting D, the exhausted liquid is drained through a purge. In the upper zone of D, the relatively warm gaseous mixture (CO + CO_2_, at about 55 °C), is collected, evacuated and sent towards the depressurizer DP_2_. This one releases the pressure from 2 bar to 1.2 bar (with a slight endothermic reaction). From here, the gas reaches the furnace (F), to convert CO to CO_2_ (through combustion) and produce hot gases. The desorber contains a distribution zone of the absorbent liquid, also known as a spraying zone, which achieves the finest dispersion of that liquid. The fine drops fall into a filling area, where the desorption of CO from the absorbent liquid is completed. The distributor also is equipped with an external electrical resistance (outside the desorber), which raises the temperature of the absorbent liquid from 25–30 °C to 55–60 °C, in the initial phase of plant operating. 

#### 2.2.5. The Iron Reactor (IR)

The iron reactor (IR) is the second key component of HYRON plant. It can work as a stand-alone installation. This equipment is quite complex and exhibits innovative constructive elements. As mentioned in Section 2, the s-Fe-p production technology uses pure shredded iron ore Fe_2_O_3_ as raw material (with granularity of 20–250 μm), purchased from the steelmaking industry. The ore processing is carried out vertically, after loading the IR at the top, with a flow of approximately 29 kg/h. To increase the process efficiency, the redox reaction is carried out at a temperature around 572 °C. As already known, the reduction performed by means of hydrogen is incomplete at temperatures below 565 °C and over 580 °C. Therefore, the ore is preheated at about 580 °C, by means of a plate pre-heater, which is fed with hot gases coming from F. Due to the fine granulation of the ore and the small distance between the plates, the heat transfer is relatively fast. The pre-heated ore is sustained and dispersed by means of a complex sieving system, consisting of two fixed sieves and one sliding sieve, electrically powered. Thus, the ore flow is adjusted by the speed of the mobile sieve. The two other sieves, located above and below the mobile one, are designed to reduce the ore dissipation away from the mobile sieve. This assembly allows passage through the sieve of all grains with a diameter up to 250 μm. 

Successively, the ore is sieved again through a similar system, which allows accumulating a layer of fine iron ore with thickness between 20 and 30 mm. In this area, there is an additional isolated electrical heating system (with resistors made of Kanthal strips), which aims to sensibly tune the ore temperature and to keep it at an optimum value of 570 ± 3 °C. Thus, iron oxide reduction and s-Fe-p formation are provided in a minimum reaction time (about 1–2 min). The ore layer is crossed by the flow of hot hydrogen coming from GR, as well as from the next block, a gas recycler with heat exchanger and washer (GRHEW) (as described within the next subsection). The hot hydrogen is introduced at IR bottom, completing the redox reaction (5). This reaction is produced by the physical contact between two opposite flows: of hydrogen (at a temperature of about 570 °C) and of iron ore (heated at the same temperature). The oxidation of hydrogen releases about 88% of the necessary heat for the reaction to complete. To simplify the technological process, the remaining 12% are electrically supplied by the Kanthal-based heater, carefully designed in this aim. Beneath the second sieving group, there is another free space, where the reduced ore particles are also dispersed. 

The two free spaces at the bottom of sieving systems contribute to the organization of the vertical circulation of the gaseous mixture of H_2_ + H_2_O^vapor^, generated during the cyclic oxidation reaction. As mentioned above, the preheated ore at 580 °C meets H_2_ (coming from the outside through a pipe distributor) at IR bottom. After completion of reaction (5), the H_2_ + H_2_O^vapor^ mix is evacuated at IR top. This mixture is not clean (as it captures some s-Fe-p with granularity below 40 μm) and goes to GRHEW block. The main goal of this auxiliary block is to extract the water and separate the (dried) H_2_, which will be recycled in IR as a reducing agent, together with the fresh H_2_, provided by GR. The mixture of fresh and recirculated hydrogen is firstly preheated at about 580 °C. Then, it is horizontally introduced in the mass of the reduced iron ore (more precisely, about 15 mm below its free level), through the distribution pipes network, which uniformly spread it at output of ore bulk. Consequently, the deoxidation process eventually completes and carries on continuously. As H_2_ is passing through the ore, the balance diagram of the iron oxide reduction shows that, at 572 °C, only 21% of H_2_ is converted to H_2_O [45]. To be sure, one assumes next that only 19% of H_2_ react, while the remaining 81% are not oxidized. 

The s-Fe-p, with initial temperature of 570 °C, keeps descending and passes through a cooler immersed in the powder bulk, which has a similar construction with the ore pre-heater. As a cooling agent, gases at approximately 80 °C provided by the ore pre-heater are employed. The s-Fe-p keeps cooling down to 35 °C in a water-based HE, placed at installation bottom, so that the resulted warm water can easily be purged. Now, the cooled s-Fe-p is collected in a container of 20 kg, located beneath the IR, connected to the room to reduce pyrophoricity (RRP) (described in Section 2.2.7).

#### 2.2.6. The Gas Recycler with Heat Exchanger and Washer (GRHEW)

This block includes some specific constructive elements to chemical industry. It has a modular, layered, configuration, with a HE at the top and a humidifier (a gas washer, actually) at the bottom. From IR, the mixture of 81% H_2_ and 19% H_2_O^vapor^ (at a flow of 48 m^3^_n_/h H_2_ together with 3 m^3^_n_/h H_2_O^vapor^) and some residual s-Fe-p, with small particles below 20 μm enters the top of GRHEW block. In a first stage, this mixture is cooled with combustion gas or atmospheric air from 580 °C to 150 °C. In the second stage, the mixture is further cooled to 20 °C, with an intake of cold water (possibly added with ice or snow). Here, the condensation of water vapor occurs too. The condensed water (possibly with water from ice/snow) and the dust collected from the ore are eliminated, and the dry H_2_ (at 20 °C) must be reheated in HE, by means of the gas mixture supplied by IR. The condensate and the residual s-Fe-p form a mud, which can later be used as purging material for the wastewater, leached or even as a fertilizer for the growth of algae biomass. The cold hydrogen is then immersed into a gas washer, where there is a small flow of washing water, which is totally cleaning the mud. The purified hydrogen (h-p-H_2_) enters the compressor C_3_, which recirculates it in GRHEW, in order to receive again the heat that was initially given by the above mentioned mixture. The h-p-H_2_ inputs the HE bottom (of GRHEW) with a temperature of 20 °C and outputs the HE top with a temperature around 570 °C. Next, the hydrogen flow coming from GRHEW is combined with the H_2_ flow coming from GR and completes the total flow of 60 m^3^_n_/h, which enters IR, in the reaction zone between H_2_ and iron ore. 

A mixture of 48 m^3^_n_/h H_2_ and 15 m^3^_n_/h H_2_O^vapor^ enters the HE, at the temperature of 580 °C. From the 15 m^3^_n_/h H_2_O^vapor^, the amount of 12 m^3^_n_/h results from the ore deoxidation with hydrogen, whereas 3 m^3^_n_/h are made of recirculated steam, due to the incomplete removal of water from hydrogen. From the mixture, by the final cooling down to 20 °C, the flow of 48 m^3^_n_/h H_2_ and 3 m^3^_n_/h H_2_O^vapor^ results. However, before reaching the washing zone (beneath HE), the mixture is cooled to 150 °C, while the given heat is gradually taken by the cold ascending hydrogen (from HE bottom to the top). The heat flow exchanged between the thermal agents is estimated to about 9,000 kcal/h. The hot hydrogen (at 570 °C) sent to IR is mixed with other 12 m^3^_n_/h H_2_ provided by GR. The total flow of 60 m^3^_n_/h H_2_ constitutes the reducing agent of the iron ore. 

A flow of 48 m^3^_n_/h H_2_ + 3 m^3^_n_/h H_2_O^vapor^ passes through a water drop separation plan, which is designed to prevent mixing between hydrogen and water drops. The hydrogen is then evacuated by means of compressor C_3_ connected at HE bottom. 

#### 2.2.7. The Room to Remove Pyrophoricity (RRP)

This block also is referred to as s-Fe-p collector and exhibits quite a simple structure, being connected to IR bottom. Once the fresh s-Fe-p has reached the IR bottom by gravitational fall, it is collected into the RRP at a temperature of about 80 °C, with a flow of 20 kg/h. During the s-Fe-p harvest, a void pump (VP) is employed to achieve a pressure of 1 mbar at most. Its purpose is to exhaust the molecular and/or atomic hydrogen, which can superficially be absorbed by the s-Fe-p. At the same time, the s-Fe-p is cooled when the VP is shutting down and an amount of argon (Ar) is admitted in the precinct (after 15 min). The argon replaces the hydrogen and the potential water vapor, in order to prevent the subsequent redox reactions between the iron and the atmospheric air. The iron with (practically) no pyrophoricity at all (i.e., the final inert s-Fe-p) can subsequently be packed in any form, with no problems of manipulation. 

### 2.3. On the Design of HYRON Installation Main Blocks

#### 2.3.1. Designing the Furnace (F)

The furnace is sized to burn gases with a flow of approximately:

12.87 m^3^_n_/h CO + 3 m^3^_n_/h H_2_ + 0.3 m^3^_n_/h CO_2_ + 0.1 m^3^_n_/h N_2_ + 1.0 m^3^_n_/h H_2_O. (6)

After some computations, it resulted that the total heat flow is of 48,320 kcal/h or about 56.2 kW, from which only 7,977 kcal/h or about 9.2 kW are used within the GR (see the next section). This energy waste was accepted only in the aim of greening the relatively large (and very toxic) CO production, by burning the entire oversupply. In order to maximize the thermal inertia of the furnace, its walls were coated with ceramic fibres, able to resist up to 1200 °C. This solution increases the installation efficiency, especially in case of frequent restarts. The furnace has a vertical cylindrical shape with the following sizes: inner diameter of 0.5 m, inner height of 1 m, total wall thickness of 0.15 m, outside diameter of 0.8 m, total height of 1.3 m. In order to prevent hazards, the furnace is controlled by a thermocouple, which limits the upper temperature to 1050–1100 °C. 

#### 2.3.2. Designing the Gas Reactor (GR)

One aims to obtain 20 kg/h s-Fe-p (for which a flow of 0.6 m^3^_n_ H_2_/kg Fe is required) and at least 12 m^3^_n_/h H_2_. To reach for this goal, it is necessary that HYRON installation produces a higher flow of hydrogen, e.g., in amount of 15 m^3^_n_/h H_2_ (including the hydrogen coming along with CO_2_ and CO).

The chosen raw material is a ligno-cellulosic waste type of biomass (preferably sawdust), since it recycles CO_2_ in the natural photosynthesis process, is abundant, inexpensive, clean and it has a hydrogen content superior to other solid fuels. The mass composition of this biomass is similar to that of hardwood or resinous and straws. More specifically, a rudimentary proximate analysis led to the following composition of acquired sawdust: 50.8% carbon, 6.2% hydrogen and 43% oxygen. The participation of nitrogen (0.6%) was neglected and a minimum absolute humidity of 3% was considered. It follows that 1 kg of dried cellulosic waste (e.g., dsd) contains 42.3 mol carbon, 31 mol hydrogen and 13.4 mol oxygen.

As reported into the literature [45,46,47,48], at a temperature of 1000 °C, when biomass gasification occurs (according to reaction (1)), the syngas has the volumetric concentrations of 48% CO (41 mol CO), 1.5% CO_2_ (1.3 mol CO_2_), while 42.3 mol C are fully included into CO and CO_2_. The carbon oxidation requires 21.8 mol O_2_, but the biomass contains only 13.4 mol O_2_. Thus, the remaining 8.4 mol are obtained from 16.8 mol H_2_O. Consequently, the gasification reaction, in mol, is:

42.3 C + 31 H_2_ + 13.4 O_2_ + 16.8 H_2_O = (31 + 16.8) H_2_ + 41 CO + 1.3 CO_2_. (7)

Since only a part of water vapor participates to the useful oxygen release, a water supplement is necessary, in order to keep the reaction alive. By only adding 3 mol of water to both terms of Equation (7), one obtains:

42.3 C + 31 H_2_ + 13.4 O_2_ + 19.8 H_2_O = 47.8 H_2_ + 41 CO + 1.3 CO_2_ + 3 H_2_O, (8)

while the overall balance of water concentration is preserved. 

For the useful water consumption (avoiding the steam production), the moisture of gasified sawdust has to be of 0.35 kg water within each kg of wsd, which means about 0.26 kg water within each kg of dsd. Hence, 1 kg dsd = 0.26/0.35 kg wsd **≅** 0.7429 kg wsd. The gasification of dsd, by using Equation (8), produces 1.07 m^3^_n_ H_2_ / kg wsd **≅** 0.7949 m^3^_n_ H_2_ / kg dsd. 

In the first term of Equation (7), direct measurements have shown that the heat intake is limited to 4800 kcal/kg wsd. In the second term, the heat is converted from H_2_ and CO energy to the amount of approximately 4474 kcal/kg wsd. Nevertheless, gasification will only occur if the total heat intake equals the total transferred heat. Therefore, the difference of 326 kcal/kg wsd has to be supplied by the furnace. Obviously, the furnace has to ensure the necessary energy to produce at least 12 m^3^_n_/h H_2_ to send to the IR. To increase the purity of hydrogen employed to produce s-Fe-p, 13.4 m^3^_n_/h H_2_ has to be generated, the supplement of 1.4 m^3^_n_/h H_2_ being estimated as sufficient for a higher purification. The caloric efficiency of GR can easily be estimated, as this block is partly self-sustainable: it produces 4474 kcal/kg wsd and requires 4800 kcal/kg wsd, i.e., with 326/4474% **≅** 7% more. The GR efficiency is thus of about 93%, theoretically. Practically, after building the GR, its efficiency has been estimated to about 90%. 

All the heat is required for cellulosic waste drying, vaporisation and overheating of supplementary water, pyrolysis and full gasification, increasing the reactants temperature up to 1000 °C, various dissipations (estimated at 75 kcal/kg wsd), as well as for the transfer outside the installation. Drying the cellulosic waste at about 100 °C consumes 281 kcal/kg wsd, and heating up to 450 °C, in order to complete the pyrolysis, consumes 273 kcal/kg wsd. 

The heat consumption of pyrolysis and gasification is included in the isothermal balance of the two sides in Equation (8). The heating at 1000 °C of syngas resulted during the wsd evolution consumes about the same heat it gives during the cooling at 450 °C. The cooling between 450 °C and 100 °C is practically achieved by contributing to the reheating of the nearly dried syngas from about 20 °C to almost 950 °C (the water being drained in the liquid phase). 

One can set as target a flow of 15 m^3^_n_/h H_2_ to be generated by the GR. This involves a consumption of approximately 14 kg/h dsd (with 26% humidity) or, equivalently, of about 19 kg/h wsd (with 35% humidity). Moreover, the hydrogen sent to IR transfers a heat flow of about 3413 kcal/h. Then, the total energy the GR has to consume is:

326 × 14 + 3413 = 7977 kcal/h ≅ 9.28 kW.(9)

In F enters a flow of 41 mol/h CO and 3 m^3^_n_/h H_2_, which carry 48,320 kcal/h. Since the GR consumes only 7977 kcal/h, during the experiments, one decided to evacuate in the atmosphere the supplement of:

48,320 − 7977 = 40,343 kcal/h ≅ 46.92 kW,(10)

after complete burning of CO (to avoid any risk). 

As already mentioned in Section 2.2.2, the intensification of the heat exchange in the GR was achieved by radial blades placed on the inner mobile cylinder. In order to design the GR, a tester with electric heating for the two cylinders was first built. The drive blades had initially a length of 20 mm. Later on, after several tests, the GR module with gas heating was built. In both cases, the value of the heat transfer coefficient reported on the cylinders was of 325 kcal/m^2^/h/K. By increasing the blade length to 30 mm, this value jumped by over 40%, at 450 kcal/m^2^/h/K. Additionally, the thermal conductivity of blades has been amplified, by making them of copper (they can also be made of aluminium or latten in this aim).

Since the heat transfer is simultaneously realized by conduction and convection, it is natural to analyze both phenomena, in order to obtain the right size of GR. 

Considering the mobile cylinder speed of 0.2 m/s and a rib tilting of 6°, it results a theoretical radial moving speed of sawdust estimated at 0,1xtg(6°) **≅** 0.02 m/s. The ribs take over the hot sawdust and move it to the central zone, stirring it continuously and recirculating it between the central cold zone and the hot lateral zone. If the real moving speed would be only 20% of the theoretical one, i.e., 0.004 m/s, since the density of the sawdust is of 200 kg/m^3^, the specific flow of sawdust moved from the outer heater fixed cylinder to the inner colder mobile cylinder can be calculated as follows:

0.5 × 0.004 (m/s) × 3600 (s/h) × 200 (kg/m^3^) ≅ 1440 kg/m^2^/h. (11)

In Equation (11), the factor 0.5 is required by the existing of the sawdust reverse flow, from the cold cylinder to the hot cylinder. 

Considering the specific heat of the sawdust of 0.6 kcal/kg/K and aiming that the warm wall transfers heat with a specific flow over 5000 kcal/m^2^/h, it results that the difference between the temperature of the sawdust near the lateral wall and the temperature of the central mixture is ΔT = 5000/1440/0.6 K **≅** 5.8 K. The conductive intake is estimated at about 20% of the moving sawdust intake, so that ΔT is reduced to 0.2 × 5.8 K **≅** 1.2 K. It results a specific heat flow transferred by conduction of 1440 × 0.6 × 1.2 kcal/m^2^/h **≅** 1037 kcal/m^2^/h, which means a specific heat transfer coefficient of 1037 kcal/m^2^/h/K. 

Since the heater agent consists of the combustion gases, which produce low intensity convection under normal conditions, with a heat transfer coefficient of no more than 20 kcal/m^2^/h/K, it is also necessary to intensify this heat exchange. In the areas with temperatures below 400 °C, where the intensity of thermal radiation is relatively small, an intensification method could be the following: the gases will pass through a space between two parallel metallic plates, interconnected by thin copper wires (with maximum 1.5 mm in diameter and about 5 mm step) and blackened by pre-oxidation. The wires receive the heat with a heat transfer coefficient of approximately 100 kcal/m^2^/h/K, even at moderate speeds. They can send the heat by conduction to the walls that separate the gas channels, of 15 mm wide each. This increases the convective heat transfer coefficient to the value of 150 kcal/m^2^/h/K. 

With this solution of convection heat transfer intensifying, an average heat transfer coefficient can be estimated, as follows:

1/k_med_ = (1/1037 + 1/150) m^2^.h.K/ kcal ⇒ k_med_ ≅ 131 kcal/m^2^/h/K. (12)

Then, according to Equation (12), the average temperature offset is: ΔT_med_ = 5000/131 K **≅** 38 K.

The surface of the heater cylinder is estimated to the value of 7949/5000 m^2^ **≅** 1.6 m^2^. By taking into account the average temperature offset ΔT_med_, one can choose a distance of 0.04 m between the two metallic cylinders of GR. Correspondingly, the mobile inner cylinder could have the diameter D_m_ = 0.3 m and the wall thickness of 0.003 m. Then the inside diameter of the fixed outer cylinder is D_f_ = D_m_ + 2 × (0.04 + 0.003) mm = 0.386 m. To increase the mechanical stiffness of the two cylinders, each one of them has been welded to a second coaxial cylinder. The mobile cylinder match has a smaller diameter, of 0.264 m, while the fixed cylinder match has a larger diameter, of 0.422 m. Thus, the two cylinders are welded at a distance of 0.018 mm from each other, in both cases. To estimate the height h of the cylinders, the Equation below can be used:

1.6 m^2^ = 2 × π × h × D_f_, (13)

as the thermal transfer surface belongs to the fixed cylinder. From Equation (13), one obtains h **≅** 0.66 m. For safety reasons, the height was chosen to h = 0.85 m. All the cylinders were built from refractory steel pipes, with close sizes to the designed ones.

The combustion gases circulate through GR at a speed of about 0.21 m/s. In the hot part (at approximately 1000 °C), they enter with 1 m/s speed and in the cold part (at 80 °C), the burnt gases are evacuated at 0.28 m/s speed. The GR is thermally insulated, according to the temperatures of the outside cage (between 100 °C on top and 1000 °C at bottom). A ceramic coating is used to protect GR materials from thermal expansion during high operating temperature and ensures the insulation. Its thickness varies between 0.05 m on top and 0.12 m at bottom. The outer diameters of GR are approximately of 0.49 mm on top and of 0.63 m at bottom.

#### 2.3.3. Designing the Cooler (C)

As already mentioned in Section 2.2.2, in normal functioning regime, the RG produces a hot syngas consisting of H_2_, CO, H_2_O^vapor^ and small amount of CO_2_. The hydrogen separation requires preliminary cooling of syngas, from approximately 1000 °C to 20 °C. As a compact and economical solution, the C was built from 1.5 mm thick plate iron (refractory in the hot zone), with rectangular channels of only 1.5 mm in width, in order to increase convection. The heat transferred by the cooled syngas (from about 1000 °C to 20 °C) is almost completely recovered by reheating the freshly separated h-p-H_2_. 

In the sequel, the C sizing and the estimation of the heat transfer surface are presented, based on the heat capacities of gases. The average temperature of syngas is (1000 + 20)/2 °C = 510 °C = 783 K. Consequently, the specific volumetric heat capacities of every gas in the mixture are easily specified from tables: $c_{{v,H}_{2}}\;=\;0.3124$ kcal/m^3^/K, $c_{v,\mathrm{CO}}\;=\;0.3242$ kcal/m^3^/K, $c_{{v,\mathrm{CO}}_{2}}\;=\;0.4875$ kcal/m^3^/K, $c_{{v,H}_{2}O}\;=\;0.3857$ kcal/m^3^/K (both under and over 100 °C). 

The average specific heat capacity of the gaseous mixture is derived from Dulong-Petit Law:

(14) $$c_{v}=\sum_{k}r_{k}\cdot c_{v,k}$$

where $c_{v,k}$ is the specific volumetric heat capacity of the chemical k and $r_{k}$ is its mass ratio. The mass ration of each syngas compound has been estimated in Section 2.2.3. Thus, the Equation (14) leads to the average syngas heat capacity: 

44.1% CO; 2.1% CO_2_; 2.5% H_2_O^vapor^

(15) $$c_{v}=0.513\;c_{{v,H}_{2}}+0.441\;c_{v,\mathrm{CO}}+0.021\;c_{{v,\mathrm{CO}}_{2}}+0.025\;c_{{v,H}_{2}O}≅0.3231\;\mathrm{kcal}/m^{3}/K.$$

The syngas total flow F= 28.7 m^3^/h, the water vaporization rate is $\nu_{H_{2}O}\;=\;10.45$ kg/h and the water vaporization heat capacity is $\lambda_{H_{2}O}\;=\;1.4444$ kcal/kg. Based on these specifications, it is possible to compute the heat flow transferred by the syngas as below:

(16) $$Q_{\mathrm{syngas}}=F\cdot c_{v}\cdot \Delta T_{\mathrm{syngas}}+r_{H_{2}O}\cdot \nu_{H_{2}O}\cdot \lambda_{H_{2}O},$$

where the difference of the two temperatures is $\Delta T_{\mathrm{syngas}}={\left(1000-20\right)}^{o}C=980^{o}C=980\;K$, and $r_{H_{2}O}=0.025$. It results $Q_{\mathrm{syngas}}≅9088$ kcal/h. This heat must be received by a flow of hydrogen with 95% purity at least, heated from 20 °C to 800 °C (hence, with a temperature difference of $\Delta T_{H_{2}}=780^{o}C=780\;K$). Given the hydrogen gaseous flow of about $F_{H_{2}}=0.513\;F≅14.7$ m^3^/h, the heat is received by hydrogen with the flow:

(17) $$Q_{H_{2}}=F_{H_{2}}c_{{v,H}_{2}}\Delta T_{H_{2}}≅3582\;\mathrm{kcal}/h.$$

The hydrogen that C supplies is combined with the recirculated hydrogen provided by GRHEW (at 50 °C), in order to obtain a hydrogen flow with a temperature around 670 °C, at IR input. The hourly heat difference, $\Delta Q=Q_{\mathrm{syngas}}-Q_{H_{2}}=9088-3582=5506$ kcal/h, is received by a water flow, with the temperatures: $T_{{\mathrm{in},H}_{2}O}=20^{o}C=293\;K$ (at input), $T_{{\mathrm{out},H}_{2}O}=90^{o}C=363\;K$ (at output). This leads to a water flow of: $\nu_{H_{2}O}=\Delta Q/c_{{m,H}_{2}O}/\Delta T_{H_{2}O}=78.66$ kg/h, where $c_{{m,H}_{2}O}=1$ kcal/kg/K is the specific mass heat of water. In order to decrease the final temperature of cooling water, this flow can be increased to the value of $\nu_{H_{2}O}=700$ kg/h. The final temperature will be then:

(18) $$T_{{\mathrm{out},H}_{2}O}=T_{{\mathrm{in},H}_{2}O}+\frac{\Delta Q}{c_{{m,H}_{2}O}\cdot \nu_{H_{2}O}}=\left(293+\frac{5506}{1\cdot 700}\right)\;K≅301\;K={28\;}^{\circ }C,$$

which is a realistic value. 

Constructively, a pipe-in-pipe type of C was selected. This means that the cooling agent flows inside the inner pipe, and the gases to be cooled flow inside the outer pipe, which surrounds the inner one. There are two cooling zones: one for hydrogen and another one for water. For both of them, the cooling pipes have to be sized appropriately. 

For hydrogen, it is necessary to start from the following design data specific to the block A-HE-D: output flow from A of 14.7 m^3^_n_/h; temperature of 20 °C; output pressure from A of 2 bar; input pressure in DP_1_ of 1.2 bar; gaseous mixture speed of 8 m/s. It results that the exhausting pipe diameter of H_2_ in DP_1_ could be computed as follows:

(19) $$D_{{\mathrm{in},H}_{2}}=\sqrt{\frac{4}{\pi }\times \frac{2}{1.2}\times \frac{14.7}{8\cdot 3600}}\;m≅0.03\;m$$

The same diameter is chosen also for the inner pipes of H_2_ inside C. Likewise, in order not to complicate the construction of C, the water pipes can have the same diameter, $D_{{\mathrm{in},H}_{2}O}=D_{{\mathrm{in},H}_{2}}$. For all of these pipes, the thickness was set to 2 mm. The outer pipes could have the diameter: $D_{{\mathrm{out},H}_{2}O}=D_{{\mathrm{out},H}_{2}}=2\cdot D_{{\mathrm{in},H}_{2}}=0.06\;m$.

The length of a H_2_ pipe, $L_{H_{2}}$, was estimated based on the value of heat transfer flow between gases leaving the GR and hydrogen ($Q_{H_{2}}≅3582$ kcal/h, Equation (17)). This flow is radially transmitted through the cylindrical wall of the pipe, being defined by:

(20) $$Q_{H_{2}}=K\cdot \bar{S}\cdot \bar{\Delta T},$$

where K is the total heat transfer coefficient, $\bar{S}=2\pi \;\bar{r}\;L_{H_{2}}$ is the average surface of heat transfer, estimated by means of the logarithmic mean radius of the pipe, $\bar{r}$, and $\bar{\Delta T}$ is the average temperature difference. By definition, $\bar{r}=\left(r_{\mathrm{out}}-r_{\mathrm{in}}\right)/\log\frac{r_{\mathrm{out}}}{r_{\mathrm{in}}}$, where $r_{\mathrm{in}}=D_{{\mathrm{in},H}_{2}}/2=0.015$ m is the inner radius, while $r_{\mathrm{out}}=0.017$ m is the outer radius of the pipe. Thus, $\bar{r}=0.016$ m (same as the arithmetic mean) and $\bar{S}≅0.1\times L$ [m^2^]. Next, for syngas coming from GR at the temperature of 1000 °C = 1273 K, one can evaluate the output temperature at the output of C, after being cooled with the cold hydrogen coming from DP_1_. Thus,

(21) $$T_{\mathrm{out},\mathrm{syngas}}=1273\;[K]-\frac{Q_{H_{2}}}{F_{H_{2}}c_{{v,H}_{2}}\;}=\left(1273-\frac{3582}{14.7\times 0.3124}\right)\;K≅493\;K={220\;}^{\circ }C.$$

Similarly, the cold hydrogen entering C has a temperature of 20 °C = 293 K, and the hydrogen entering IR has the temperature of 800 °C = 1073 K. Hence,

(22) $$\bar{\Delta T}=\frac{1}{2}\left(\frac{1273+493}{2}-\frac{293+1073}{2}\right)\;K=100\;K.$$

To evaluate the transfer surface, firstly the constant K has to be derived, from the Equation below:

(23) $$K=\frac{1}{\frac{2}{\alpha }+\frac{D_{{\mathrm{out},H}_{2}}}{\lambda_{p}}},$$

where α is a partial heat transfer constant, $D_{{\mathrm{out},H}_{2}}=2\;r_{{\mathrm{out},H}_{2}}=0.034$ m is the outer pipe diameter, and $\lambda_{p}=46.5$ W/m/K is the thermal conductivity of the pipe. In order to calculate the constant α, the Nusselt number (Nu) is used, which leads, on one hand, to: $\alpha =\frac{\lambda_{p}\mathrm{Nu}}{D_{{\mathrm{out},H}_{2}}}$. On the other hand, this criterion can be determined by using Donahue’s Equation:

(24) Nu=0.24×Re0,6⋅Pr0,33(μμp)0,14,

where Re is the Reynolds number, Pr is the Prandtl number, μ and $\mu_{p}$ are the dynamic viscosities of the fluid in the middle of the flow and at the wall, respectively. Usually, the last ratio in Equation (24) is approximately unit. In order to estimate the two dimensionless numbers (Re and Pr), one has to know: the hydrogen flow speed through the pipes ($v_{H_{2}}=8$ m/s), the cinematic viscosity of hydrogen $\upsilon_{H_{2}}≅1.709\times 10^{-4}$ m^2^/s and the hydrogen density $\rho_{H_{2}}=0.08961$ kg/m^3^. Thus:

(25) Re=vH2Dout,H2υH2=8×0.0341.709×10−4≅1591,6;Pr=cm,H2ρH2υH2λp=14,592.6×0.08961×1.709×10−446.5≅0.0048

taking into account that, for hydrogen, the specific mass heat is:

(26) $$c_{{m,H}_{2}}=\frac{c_{{v,H}_{2}}}{\rho_{H_{2}}}=\frac{0.3124}{0.08961}\;\frac{\mathrm{kcal}}{\mathrm{kg}.K}=4185.8\times \frac{0.3124}{0.08961}\;\frac{J}{\mathrm{kg}.K}≅14,592.6\;\frac{J}{\mathrm{kg}.K}$$

According to Equations (24) and (25), the Nusselt number is: Nu≅3.4365. Consequently, α≅4700 W/m^2^/K and K≅864.52 W/m^2^/K (see Equation (23) again). Finally, from Equation (20), one obtains the pipe length:

(27) $$L_{H_{2}}=\frac{Q_{H_{2}}}{2\pi \;\bar{r}\;K\;\bar{\Delta T}}=\frac{1.163\times 3,582}{2\pi \times 0.016\times 864.52\times 100}\;m≅0.479\;m.$$

The above reasoning can be resumed for water as cooling agent, to compute the length of corresponding pipes. This time, the following water specifications have to be used: $Q_{H_{2}O}=\Delta Q=5506$ kcal/h; $\bar{r}=0.016$ m (same as for hydrogen); syngas needs to be cooled from 220 °C = 493 K to 20 °C = 293 K; the water has the input temperature of 20 °C = 293 K and the output temperature of 28 °C = 301 K (according to Equation (18)); the flow speed through the pipes is $v_{H_{2}O}=4$ m/s; the specific mass heat is $c_{{m,H}_{2}O}=1.6156$ J/kg/K; the cinematic viscosity (at the average temperature of 24 °C) is $\upsilon_{H_{2}O}≅9.131\times 10^{-7}$ m^2^/s; the density is $\rho_{H_{2}O}=999.3$ kg/m^3^. Having these data, it results $\bar{\Delta T}=48\;K$; Re≅148,943; Pr=0.0000317; Nu=9.986; α=13,657 W/m^2^/K; K=1139.4 W/m^2^/K. Consequently, the water pipe length is:

(28) $$L_{H_{2}O}=\frac{Q_{H_{2}O}}{2\pi \;\bar{r}\;K\;\bar{\Delta T}}=\frac{1.163\times 5506}{2\pi \times 0.016\times 1139.4\times 48}\;m≅1.165\;m$$

Taking into account the obtained values, the pipe constructive lengths can be chosen as: $L_{H_{2}}=0.5\;m$ and $L_{H_{2}O}=1.2\;m$.

#### 2.3.4. Designing the A-HE-D block

A. The absorber (A)

In order to verify the results of the previous subsection, the diameter of syngas feed pipe entering A can be evaluated. As already mentioned, the warm syngas exits C with the flow $F=\;F_{C}=28.7$ m^3^/h, while the pressure equals 1 bar (before entering the compressor C_2_). By accounting the mass ratios of each compound gas, this flow can be expressed as follows:

(29) $$F_{C}=(\underset{r_{H_{2}}}{\underbrace{0.513}}+\underset{r_{\mathrm{CO}}}{\underbrace{0.441}}+\underset{r_{{\mathrm{CO}}_{2}}}{\underbrace{0.021}}+\underset{r_{H_{2}O}}{\underbrace{0.025}})F≅(\underset{F_{H_{2}}}{\underbrace{14.7}}+\underset{F_{\mathrm{CO}}}{\underbrace{12.7}}+\underset{F_{{\mathrm{CO}}_{2}}}{\underbrace{0.6}}+\underset{F_{H_{2}O}}{\underbrace{0.7}})\;{m^{3}}_{n}/h.$$

After the compressor C_2_, the flow of the gaseous mixture is calculated by applying Boyle-Mariotte Law: p.V = constant. Considering that the gases are almost ideal, it results that, at a pressure of 2 bar, the overal flow becomes $\;F_{A}=F_{C}/2=28.7/2=14.35$ m^3^_n_/h. If the optimum speed of the syngas is 8 m/s (as of hydrogen through C), the minimum diameter of the feeding pipe in A is derived:

(30) $$D_{\mathrm{syngas},A}=\sqrt{\frac{4}{\pi }\frac{F_{A}}{8}}=\sqrt{\frac{4}{\pi }\times \frac{14.35}{8\times 3600}}\;m≅0.0252\;m$$

For safety reasons, the diameter can be chosen as for C, i.e., $D_{\mathrm{syngas},A}=0.03\;m$. The same diameter is also chosen for the discharge pipe of the unabsorbed hydrogen in A, on the top. It will have a pressure of about 2 bar, a temperature of maximum 35 °C and will enter a depressurizer to bring it to the pressure of 1.1–1.2 bar. The depressing process is strongly endothermic, such that the hydrogen becomes highly dry and cold (at approximately 25 °C). If the depressurizer is vertically placed, any wet compounds of hydrogen will fall back into A. 

Further, the absorption process is analyzed. From literature data [45,46,47,49,50], it results that in order to absorb 16 mL of CO, 1 mL of absorbent liquid is required, under normal conditions. For simplicity, the same ratio is assigned to CO_2_ gas. Thus, for a flow of about 12.7 m^3^_n_/h CO + 0.6 m^3^_n_/h CO_2_ = 13.3 m^3^_n_/h (CO + CO_2_), it is necessary an absorbent fluid flow of about 0.83 m^3^/h. To be sure, the absorbent liquid flow is set to 1 m^3^/h. This liquid enters A at the pressure of 2 bar, by means of a pump, which leads to a flow of $F_{\mathrm{al}}=0.5$ m^3^/h, by using the Boyle-Mariotte Law. For an optimum inlet speed of the liquid in A of $v_{\mathrm{al}}=0.8$ m/s, the diameter of the liquid inlet/outlet pipe is:

(31) $$D_{\mathrm{al}}=\sqrt{\frac{4}{\pi }\frac{F_{\mathrm{al}}}{v_{\mathrm{al}}}}=\sqrt{\frac{4}{\pi }\times \frac{0.5}{0.8\times 3600}}\;m≅0.01487\;m$$

In order to use the same type of pipe to as many components of the HYRON installation, one chooses the same diameter as above, i.e., $D_{\mathrm{al}}=0.03\;m$. 

The efficiency of gas absorption can be improved by increasing the mass transfer surface of certain gases in a liquid, through fillers. Consider that we have a 1 m^3^ room full of fillers. After several experiments with different types of filler, it has been found that an optimal absorption surface is obtained by using cylindrical grains of polyamide 66 with 43% glass fibers having the size $D_{g}=h_{g}=3$ mm and density $\rho_{\mathrm{fill}}=862.5$ kg/m^3^. The measured mass of the grains in the room is $m_{\mathrm{fill}}=414\;\mathrm{kg}$. It follows that the grains take the effective volume $V_{\mathrm{fill}}=m_{\mathrm{fill}}/\rho_{\mathrm{fill}}=0.48$ m^3^ (accordingly, the air volume of is $V_{0}=0.52$ m^3^). Since the surface of a grain is $S_{g}≅42.41$ mm^2^ and its volume is $V_{g}≅21.21$ mm^3^, the number of grains in the room is $N_{g}=\left\lfloor V_{\mathrm{fill}}/V_{g}\right\rfloor ≅22,630,834$. They provide the total absorption surface $S_{\mathrm{fill}}=N_{g}S_{g}≅959.77$ m^2^. In order to size the absorber, it is important to define a parameter called the nominal diameter of the filler. This is actually the diameter of a sphere of volume equal to $V_{\mathrm{fill}}$ and can be calculated as:

(32) $$D_{\mathrm{fill}}=2\sqrt[{3}]{\frac{3V_{\mathrm{fill}}}{4\pi }}≅0.9714\;m$$

To determine the apparent moving speed of gas bubbles ($v_{a}$) through the selected filler, some tests of air sparging in water were run, in a column with heights ranging from 5 to 50 cm. The apparent speed is estimated by dividing the sparged gas flow $F_{g}$ to the surface $S_{g}$ corresponding to the gas bubbles accumulated at the top of filler column. This surface is evaluated by considering that the gas bubbles are placed on a disk with a certain radius. The results of tests are synthesized in Table 1. 

**Figure 2:**
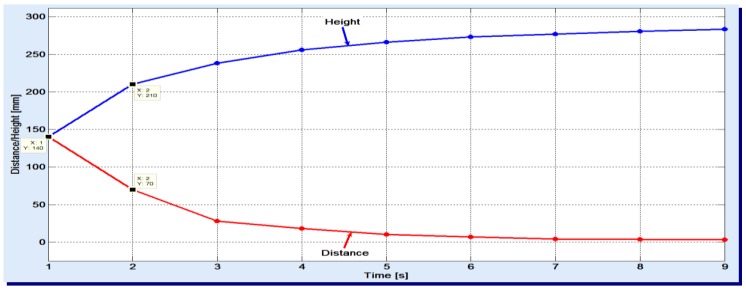
Dynamic behavior of gas bubbles passing through the absorption filler.

The used gas flow was set to the minimum necessary to stand against the hydrostatic pressure of column and complete the sparging. One noticed that the apparent speed slightly decreases with the increase of height. In the experiments, a 50 cm high filler column was used and the air was sparged. The results are displayed in Figure 2. 

Looking at the two variations, one can see, for example, that the gas bubbles have passed 140 mm in the first second and only 70 mm in the next second. As the duration increases, smaller and smaller distances are covered by the gas bubbles. These graphics also reveal that the entire filler column is basically divided into two zones:

- the lower zone, of bubbles breaking, where absorption is less present; here, the syngas meet already saturated liquid with absorbed gases;
- the upper zone, of effective absorption, with apparent speed of 3–5 mm/s.

In order to size the absorber, a known design method, which involves using the surface $S_{\mathrm{fill}}$, the diameter $D_{\mathrm{fill}}$ (of Equation (32)), Semmelbauer Law and Delong-Petit Law was employed. The method is similar to the one described at length in [51]. The volume of the filler column in the absorption process was estimated to at most 0.1 m^3^ and, therefore, for the nominal diameter of absorber column equal to 0.5 m, the minimum height of this column is of 0.55 m (by accounting the two aforementioned zones). One can choose a lower rounded value for this height, since absorption occurs to some extent in the first zone as well. However, in order to increase the absorption target rate to 99%, to allow various approximations in the technological design and to prevent the possible malfunctions while operating the installation (such as excessive temperature rising, flooding of the absorbent liquid column, etc.) the A was considered to have cylindrical shape with total height $H_{A}=0.95\;m$ and diameter $D_{A}=0.5\;m$. 

The A compartments are as follows. The inferior zone I, at A bottom, has the height $H_{A,I}=0.1\;m$. In this zone, a pipes distribution system (with the same diameter of 0.03 m) is included. Each pipe has several holes of 3 mm, allowing the gas mixture to be sparged in the A, with a pressure of about 2 bar. The zone II, of the grains filler, has the height $H_{A,\mathrm{II}}=0.5\;m$. The zone III, of feeding and liquid relaxation has the height $H_{A,\mathrm{III}}=0.2\;m$. In this zone, the recirculated liquid feeding pipe is laterally connected (in the upper side). The zone IV, of hydrogen accumulation, has the height $H_{A,\mathrm{IV}}=0.05\;m$. The zone V, of drops deflector, on A top, has the height $H_{A,V}=0.1\;m$.

B. The Desorber (D)

The desorption process is completely reversible to absorption of gases into liquids. As in the case of absorption, both a very large surface of the liquid (to quickly release the absorbed gas) and a higher temperature (to help the desorption) are required. As already known, at 58 °C, the ammonium carbonate (NH_4_)_2_CO_3_ decomposes. Therefore, a saturated solution of CO and CO_2_ that enters the desorber at 68 °C was chosen. This temperature cannot be exceeded, otherwise the ammonium can be released, together with CO_2_. 

The D block works under pressure (as A block does, as well). As already mentioned, the efficiency of the gas absorption process is estimated at 99%. The size computations are similar to those in the previous subsection. Thus, D results of cylindrical shape as well, with the height $H_{D}=0.95\;m$ and the diameter $D_{D}=0.5\;m$. 

The D compartments are as follows. In the zone I, on D top, of height $H_{D,I}=0.1\;m$, lies the drop deflector. This is identical to zone V of A. On the superior cover of D, an outlet flange to evacuate the gas mixture CO + CO_2_ is mounted. All the sizes are identical to those of A and with the same tightness requirements (especially as carbon monoxide (CO) is a toxic gas). Zone II, of saturated solution inlet, has the height $H_{D,\mathrm{II}}=0.05\;m$. To simplify the construction of the modular assembly, one can use the same configuration of distribution pipes as in zone I of A. The spraying zone III has the height $H_{D,\mathrm{III}}=0.2\;m$. In this area, finest sprays are obtained through nozzles. The filler column zone IV has the height $H_{D,\mathrm{IV}}=0.5\;m$ and is similar to zone II of A. Here, the grain sizes can vary up to 4–6 mm. The zone V, where the recirculating liquid is collected, has the height $H_{D,V}=0.1\;m$. The flow of recirculating liquid equals the one of the absorbent solution (1 m^3^/h), at the temperature of 55–58°C.

C. The Heat Exchanger (HE)

The absorption process is carried out at a different temperature from the desorption one, so that A and D are connected to each other by means of an HE (see Figure 1 again). Thus, the absorbent liquid (a copper-ammonium solution) enters HE, where is heated in the first stage from 35 °C to 65 °C and is cooled in the second stage from 55 °C to 25 °C. The heat power has the same value in both situations, and the heat capacity of recirculated liquid is the same as for the saturated liquid. The HE can be built following the classical pipe-mantle model. The heat transfer surface $A_{\mathrm{HE}}$ (of HE), can be derived from the dynamic heat transfer Equation below:

(33) $$\dot{Q}=F_{\mathrm{abs}}\cdot c_{\mathrm{abs}}\cdot \Delta T=K\cdot A_{\mathrm{HE}}\cdot \bar{\Delta T}\Rightarrow A_{\mathrm{HE}}=\frac{F_{\mathrm{abs}}\cdot c_{\mathrm{abs}}\cdot \Delta T}{K\cdot \Delta \bar{T}},$$

where $F_{\mathrm{abs}}$ (kg/s) is the mass flow, $c_{\mathrm{abs}}$ (J/kg/K) is the heat capacity, both of the absorbent liquid, ΔT [K] is the temperature difference, K (W/m^2^/K) is the total heat transfer coefficient of HE and $\Delta \bar{T}$ (K) is the average temperature difference. One can easily note that the absolute temperature difference is the same in both stages, i.e., 30 K. 

Given the following sizing values: ΔT=30 K; $K≅865\;{W/m}^{2}/K$ (estimated for the “through pipes–between pipes” type, with roughness λ=0.2); $\Delta \bar{T}=(65+35-55-25)/2=10\;K$. The mass flow and the heat capacity of the absorbent liquid have to be determined. As recommended in [45], the absorbent liquid (for CO and possibly CO_2_) is an aqueous solution of ammonium, cuprous chloride and ammonium chloride. The absorbent solution is prepared after the following recipe, presented in Table 2.

From the first column of the table, it follows straightforwardly: $F_{\mathrm{abs}}=1.016/3600=0.2822\;\mathrm{kg}/s$. From the same table, an incremental-atomic type modeling can be performed to determine the heat capacity of the copper-ammonia solution. One applies Kopp-Neumann Law [52], where the heat capacity of a chemical compound (CC) made of primary elements (PE) can be evaluated as follows:

(34) $$c_{\mathrm{CC}}=\sum_{i}n_{i}\;c_{{\mathrm{PE}}_{i}}$$

where $n_{i}$ is the mol number, and $c_{{\mathrm{PE}}_{i}}$ is the heat capacity, both for $PE_{i}$. By using the law (34), for the compounds in Table 2, one obtains:

(35) $$\left\{ \begin{array}{l} c_{{\mathrm{NH}}_{3}}=1\times 8+3\times 4.3=20.9\;\;\mathrm{kcal}/\mathrm{kg}/K≅87,483\;\;J/\mathrm{kg}/K\\ c_{\mathrm{CuCl}}=1\times 6.2+1\times 5.4=11.6\;\;\mathrm{kcal}/\mathrm{kg}/K≅48,555\;\;J/\mathrm{kg}/K\\ c_{{\mathrm{NH}}_{4}\mathrm{Cl}}=1\times 3.1+4\times 2.3+1\times 5.4=17.7\;\;\mathrm{kcal}/\mathrm{kg}/K≅74,089\;\;J/\mathrm{kg}/K\\ c_{H_{2}O}=1\;\;\mathrm{kcal}/\mathrm{kg}/K≅4186\;\;J/\mathrm{kg}/K \end{array}\right.$$

From the third coloumn of Table 2, it also results the mass ratios of compounds:

(36) $$r_{{\mathrm{NH}}_{3}}≅2.6823/41.2978≅0.06495,\;r_{\mathrm{CuCl}}≅0.04084,\;r_{{\mathrm{NH}}_{4}\mathrm{Cl}}≅0.09459,\;r_{H_{2}O}≅0.79961.$$

By using the results from Equations (35) and (36) with Dulong-Petit Law (14), one obtains:

(37) $$c_{\mathrm{abs}}=r_{{\mathrm{NH}}_{3}}c_{{\mathrm{NH}}_{3}}+r_{\mathrm{CuCl}}c_{\mathrm{CuCl}}+r_{{\mathrm{NH}}_{4}\mathrm{Cl}}c_{{\mathrm{NH}}_{4}\mathrm{Cl}}+r_{H_{2}O}c_{H_{2}O}≅18,021\;J/\mathrm{kg}/K.$$

By inserting the result (37) in Equation (33), one obtains the heat transfer surface:

(38) $$A_{\mathrm{HE}}=\frac{0.2822\times 18,021\times 30}{865\times 10}\;m^{2}≅17.64\;m^{2}.$$

Constructively, HE is a vertical cylinder with the height $H_{\mathrm{HE}}=1.2\;m$. This height is chosen so that the pipes that cross it longitudinally have the length of 1.2 m ($L_{p}$) too, as in the case of C block. Obviously, these pipes will have also the diameter 0.03 m ($D_{p}$). Then, in order to ensure the heat transfer surface (38), a minimum number of parallel pipes is required:

(39) $$N_{p}=\left\lceil \frac{A_{\mathrm{HE}}}{\pi \;D_{p}L_{p}}\right\rceil =\left\lceil \frac{17.64}{\pi \times 0.03\times 1.2}\right\rceil =156.$$

They can be distributed on a disc of the cylinder base with the diameter $D_{\mathrm{HE}}=0.6\;m$, leaving a distance of 0.012 m between them.

In order to compute the practical amount of copper-ammonium solution that resides in the A-HE-D block, it is necessary to know the volumes and spaces it floods. An acceptable approximation of this volume is obtained by only accounting the volumes of A, D and HE blocks. According to A and D sizes, the following volumes flood by the absorbent liquid are obtained: $V_{A,\mathrm{abs}}=\pi \cdot D_{A}^{2}\cdot \left(H_{A,\mathrm{II}}+H_{A,\mathrm{III}}\right)/4≅0.1374\;m^{3}$; $V_{D,\mathrm{abs}}=\pi \cdot D_{D}^{2}\cdot \left(0.1+H_{D,\mathrm{IV}}\right)/4≅0.1178\;m^{3}$ (the height of 0.1 m added to the height of zone IV of D is an approximation of the partial flood volume in the other zones); $V_{\mathrm{HE},\mathrm{abs}}=\pi \cdot D_{\mathrm{HE}}^{2}\cdot H_{\mathrm{HE}}/4≅0.4665\;m^{3}$. From here, one obtains the total volume flood by the absorbent liquid: $V_{\mathrm{abs}}=V_{A,\mathrm{abs}}+V_{D,\mathrm{abs}}+V_{\mathrm{HE},\mathrm{abs}}≅0.7217\;m^{3}$. According to Table 2, each compound fills a part of this volume, depending on the number of moles. When taking into account the density of each compound (also shown in Table 2), one obtains the corresponding quantities to mix, in order to prepare the absorbent liquid to use (see the last column of Table 2). Consequently, the overall quantity of absorbent liquid to prepare is of 815.49 kg. This recipe was selected so that the price is affordable (cuprous chloride being quite expensive).

The absorbent liquid in this recipe is obtained as follows: the water is mixed first with the two salts (CuCl and NH_4_Cl), then the ammonium (NH_3_) is added and, finally, the mix is blended until the solution becomes homogenous. The preparation is realized in a plastic made bowl of sufficient capacity. Due to its basic nature, the solution will be able to absorb CO_2_ too, this being transformed into ammonium carbonate [(NH_4_)_2_CO_3_] during the absorption process. The copper-ammonium solution has the property that 1 mL (of it) can absorb 16 mL CO. The desorption process may have lower efficiency than the absorption process. It is possible that the desorption process is less efficient than the absorption. The decomposition point of ammonium carbonate is at 58°C, which requires that the desorption process of CO and CO_2_ occurs at 60–65 °C. Therefore, the copper-ammonium solution should be refreshed periodically with 5–10% (once every 24 h), in order not to affect the absorption power. More specifically, 5–10% of the total recirculated solution is exhausted by a purge placed at A-HE-D block bottom and an equal amount of fresh solution is added through the feeder placed on top, as Figure 1 displays. 

#### 2.3.5. Designing the Iron Reactor (IR)

As previously mentioned, IR is aimed to produce sufficiently pure, decarburized s-Fe-p, by using shredded iron ore (e.g., hematite). An important goal in the design of HYRON installation was to ensure intensification of thermo-chemical processes, by uniformly distributing the reactants, mainly the ore, the hydrogen and the steam. The tasks to perform inside the IR are: realize relatively uniform temperature, inject a uniform hydrogen flow (of 0.6 m^3^_n_/kg Fe) to cross the ore layer, work with minimum ore thickness, ensure low speed of the overall flow, below the speed of particles greater than 40 μm in size (which stand for about 95% of the ore mass). 

To fulfill those tasks, a supplementary source of heat, electrically supplied, is necessary. This heat comes in addition to the heat generated by hydrogen oxidation in contact with iron ore. After some experimental tests, it was estimated that, to form 1 kg Fe by removing the metal from Fe_2_O_3_, it is necessary a heat amount of 1763 kcal (≅7.38 MJ). Since H_2_ has a burning specific heat of 57,810 kcal/kmol, and the atomic mass of Fe is 55.845 kg/kmol, according to reaction (4) (where the stoichiometric coefficients of H_2_ and Fe are 3 and 2, respectively), the thermic deficit is: 1763 – 3 × 57,810/2/55.845 kcal/kg Fe ≅ –210 kcal/kg Fe. From the total amount of 1763 kcal/kg Fe, the quantity of 210 kcal/kg Fe represents about 12%. Therefore, to produce 20 kg s-Fe-p per hour, an amount of electrical energy of 20 × 210 kcal/h = 4200 kcal/h ≅ 4.885 kW is needed. By adding 2% for power dissipation, one has to employ a power source of 5 kW. The electrical energy is dissipated inside the IR by means of three rows of Kanthal strips, which were sized so that at a nominal voltage of 220 V, they provide 5 kW of power. Hence, the global resistance of electrical heater is 220^2^/5000 ≅ 9.7 Ω. In order to achieve this value, the strips have to be disposed in several parallel resistors. Thus, two groups of resistors were interconnected in parallel, one on each half of the IR horizontal section. Each group consists of three resistors, also connected in parallel. The required resistance of a group is of 2 × 9.7 = 19.4 Ω and each resistance (from a total of six for the whole reactor) is of 3 × 19.4 = 58.2 Ω. Starting from this value, the total length of the Kanthal row (for all six resistors) was estimated at 275 m. The Kanthal strip was disposed in 191 parallel strips, with vertical width and the distance between them of 2.3 mm.

As previously mentioned in Section 3, the mixture of 81% H_2_ and 19% H_2_O^vapor^ is cooling in GRHEW, from 570 °C to approximately 15 °C. The condensation reduces the water ratio in the hydrogen flow with 2%. Hence, one obtains a ratio of 0.19/0.81 – 0.02 ≅ 0.215 m^3^_n_ H_2_O^vapor^ / m^3^_n_ H_2_. Since 1 m^3^_n_ of water vapor weights 0.79 kg, the water vapor participation is of 0.215 × 0.79 kg H_2_O^vapor^/m^3^_n_ H_2_, meaning 0.17 kg H_2_O^vapor^ / m^3^_n_ H_2_. One aims the HYRON installation to generate 20 kg/h s-Fe-p. Coming back again to reaction (4), a flow of 20 × (3 × 18.015)/ (2 × 55.845) ≅ 9.68 kg/h H_2_O^vapor^ is produced simultaneously. (Note that the atomic mass of water vapor is 18.015 kg/kmol.) Through the ore in process of deoxidation, a flow of 9.68/0.17 ≅ 56.94 m^3^_n_/h H_2_ together with an estimated water vapor flow of 0.215 × 56.94 m^3^_n_/h H_2_O^vapor^ ≅ 12.24 m^3^_n_/h H_2_O^vapor^ have to circulate. Thus, the mixture H_2_ + H_2_O^vapor^ passes through the ore, with the following output flow (at the temperature of 570 °C = 843 K):

56.94 m^3^_n_/h H_2_ + 12.24 m^3^_n_/h H_2_O^vapor^ ≅ 843 × (56.94 m^3^_n_/h H_2_ + 12.24 m^3^_n_/h H_2_O^vapor^)/273 ≅ ≅ 175.83 m^3^/h H_2_ + 37.8 m^3^/h H_2_O^vapor^ ≅ 0.049 m^3^/s H_2_ + 0.011 m^3^/s H_2_O^vapor^ ≅ ≅ 0.06 m^3^/s (H_2_+H_2_O^vapor^). (40)

Recall that, to convert 1 m^3^_n_ of gas in absolute m^3^, the gases law has to be employed: p.V/T = constant. Since the standard temperature of 1 m^3^_n_ is of 0 °C = 273 K, if the pressure is constant, the new volume depends on the ratio between the effective temperature (e.g., 843 K) and the standard temperature (273 K).

To allow completion of reduction reaction, it is necessary that the ore particles with sizes of at least 40 μm do not be captured by the exhausted gas from IR. Therefore, the flow section of the gas mixture was sized to the value 0.5 × 0.5 = 0.25 m^2^, which, according to Equation (40), leads to a flow speed of 0.06/0.25 m/s = 0.24 m/s. 

To estimate the iron ore flow that feeds IR (with no more than 200 μm in particles size), it is necessary to return to the reaction (4), where one notes that 2 mol of Fe are obtained from 1 mol Fe_2_O_3_ (ore). Since the atomic mass of oxygen is about 16 kg/kmol, it follows that the atomic mass of ore is (2 × 55.845 + 3 × 16) kg/kmol ≅ 159.688 kg/kmol. Then, to obtain a flow of 20 kg/h s-Fe-p, a necessary iron ore flow is estimated at:

20 × 159.688/(2 × 55.845) kg/h ≅ 28.6 kg/h.(41)

Thus, the IR can be fed with an iron ore flow of 29 kg/h. The IR bunker storage volume is of about 45 l, with a height of 0.25 m, and has to be supplied every 4 h with iron ore. It is suitable that the bunker includes the ore heater, which consists of 12 box type pieces, each one with the sizes of 0.5 × 0.15 × 0.15 [m]. This means the actual height of bunker+heater is 0.25 + 0.15 = 0.4 m. Such a box has an inlet connection for the combustion gases to come, of internal diameter of over 10 mm (at bottom) and an outlet connection to evacuate resulted gases (on top). The input temperature of the combustion gases is of 650 °C. They heat the boxes in contact with the iron ore and are evacuated at about 60 °C. Thus, the ore is heated from 20 °C to 580 °C. To keep the construction as simple as possible, the boxes were so designed that the hot gases circulate through channels of 4.5 × 11 (mm) in section, created along the 0.5 m length of the boxes and connected in cascade. 

Finally, all IR components (some of them are less important and not described in the paper), lead to an aggregate that could fit into a virtual cylinder with the diameter of 1.6 m and the height of 2.2 m (including the metallic support of 1.2 m height). 

#### 2.3.6. Designing the GRHEW Block

The GRHEW main purpose is to perform cooling of the mix 81%H_2_ + 19%H_2_O^vapor^ (at 580 °C = 853 K) by means of cold H_2_. Thus, water condensation occurs, followed by evacuation. Subsequently, the possible existing residual iron in the mix has to be removed by the gases washer located at GRHEW bottom. In the previous subsection, one estimated that the iron ore requires a necessary deoxidation H_2_ flow of 56.94 m^3^_n_/h. Furthermore, it has been set that the gas exhausting speed (which does not cause capturing the particles with more than 40 μm in size) is of 0.24 m/s. These parameters lead to an estimated section of vertical ascending reducing gaseous mixture flow of 56.94 × (853/273)/(3600 × 0.24) m^2^ **≅** 0.2059 m^2^. Thus, one can chose a section of 0.5 × 0.5 m^2^ **=** 0.25 m^2^. 

The whole construction of HE is metallic. From all acceptable constructive options, the parallel planar plate heater, with free distances of only 1.5 mm between plates, was chosen. Consider the laminar flow of the two thermic agents whose specific heat transfer coefficients are 225 kcal/m^2^/h/K (for H_2_) and 440 kcal/m^2^/h/K (for H_2_O^vapor^). The overall specific heat transfer coefficient, $k_{HE}$, is then equal to 1/(1/225 + 1/440) kcal/m^2^/h/K ≅ 149 kcal/m^2^/h/K. Taking into account the thermal resistance of the metal plate, the coefficient decreases to $k_{HE}=125\;\mathrm{kcal}/m2/h/K$. 

As explained in Section 2.2.6, the gaseous mixture coming from IR cools from 580 °C to 150 °C, while the cooling agent (H_2_) warms from 20 °C to 570 °C. Consequently, the difference between the average temperatures of the thermal agents is:

(42) $$\Delta {\bar{T}}_{HE}=\frac{(580+150)-(570+20)}{2}\;K=70\;K$$

Since the heat flow exchanged between agents is $Q_{\mathrm{HE}}=9000\;\mathrm{kcal}/h$ (see Section 2.2.6), it follows that the minimum surface required for this heat exchange is:

(43) $$S_{\mathrm{HE}}=\frac{Q_{\mathrm{HE}}}{k_{\mathrm{HE}}\Delta {\bar{T}}_{HE}}=\frac{9000}{125\times 70}\;m^{2}≅1.03\;m^{2}.$$

Considering the heat losses within HE, one can choose $S_{\mathrm{HE}}=1.4\;m^{2}$. The channels between the plates were sized to 150 × 500 [mm]. Given $S_{\mathrm{HE}}$, the necessary minimum number of such channels is then: 1.4/(0.15 × 0.5) ≅ 19, which means 20 plates. The plate thickness being of 1 mm (with 1.5 mm between plates), it results that the total width of the metallic case incorporating the HE is: (20 × 1 + 19 × 1.5) mm = 48.5 mm (as the lateral plates are in contact with the case). Hence, the HE is a metallic parallelepiped with sizes: 150 × 48.5 × 500 (mm). It is covered with a cylindrical insulating coating (through which various input/output heat agent pipes pass), of 0.46 m in diameter and 1 m high. 

For the gas washer, similar computations are developed, based on the thermodynamic analysis of water vapor condensation, cooling water heating (from 20 °C to 45 °C) through the contact with pre-cooled gases (at 150 °C) and the effective cooling of hydrogen. Thus, it resulted that the washer has a cylindrical shape with an inferior conic cap, having an inner diameter of 0.32 m and a height of 0.65 m. It is connected with the HE by means of an adjustment flange. Overall, the GRHEW has a height of about 1.65 m. 

#### 2.3.7. Designing the Room to Reduce Pyrophoricity (RRP)

It is known that the decarburized s-Fe-p has a bulk density of approximately 2,800 kg/m^3^. Then, 20 kg s-Fe-p takes a volume of 0.007142857 m^3^. Considering that s-Fe-p is relatively warm and the apparent density is about 1.8 times smaller, the effective volume of RRP should be of 1.8 × 0.007142857 m^3^ ≅ 0.0128571429 m^3^ at least. A stainless steel cylinder, with 0.4 m in diameter and 0.4 m in height was chosen. This offers about four times the required volume, in order to accelerate s-Fe-p cooling. 

### 2.4. Building a Preliminary Tester

Prior to HYRON experimenting, it is necessary to answer some questions concerning the efficient heat transfer among the raw material to be employed. The dsd is a good thermal insulator with an approximate conductivity of 0.05 kcal/m/h/K ≅ 1.163 × 0.05 W/m/K ≅ 0.05815 W/m/K. When humidified, this value increases more than five times. The heat generated to process the sawdust is slowly transferred to its heating, drying, pyrolysis and gasification. In order to reduce several times the volume of the sawdust gasification installation, it is necessary to establish some methods of heat transfer intensification. The following possible solutions were considered:

- the sawdust rummage in contact with the heater surface;
- transfering the heat from the basic heater surface in the sawdust mass;
- uniformization of the heat distribution inside the mass.

To accomplish these goals, a small experimental installation, called tester, was designed. The most important goal of tester was to allow practical evaluation of the total heat transfer constant between sawdust and hot surfaces. The total heat transfer constant is a key parameter for most of the chemical processes. Therefore, another motivation of tester building was to investigate methods for increasing this constant. (The obtained results after using this tester founded the design of HYRON plant components.)

Similar to the GR, the tester consists of two concentric cylinders: a fixed outer one and a mobile inner one, both equipped with blades. Electric heating was preferred, to simplify the adjustments and the measurements. The sawdust granulation was of 1–2 mm, using batches of 100 g wsd with 35% humidity. 

A first experiment was carried out with the two-cylinder installation equipped with a heating system, but just before adding the blades. The wsd was introduced on an active height of 50 mm, limited by pressboard diaphragms (to avoid uncontrolled evacuation of wsd and water vapor). A total power of 240 W was supplied, proportional to the active heater surfaces (100 W on the inside and 140 W on the outside), by using electrical resistors disposed outside of the outer metal cylinder and inside the inner cylinder. The temperatures were measured on the inner face of the outer cylinder and at half the thickness of the wsd layer. The measurements were performed during the drying of wsd at the temperature in its middle of 100–102 °C. Outside this range, the temperature evolution was fast, which allowed estimating the drying period with sufficient accuracy, more specifically, at 19 min. The temperature of cylinder walls varied between 140 °C and 152 °C, while the average difference between the sawdust and the mantle was of 46 K.

After endowing the cylinders with 36 blades (18 mobile and 18 fixed on each of the cylinders), a total power of 450 W was supplied (215 W on the inside and 225 W on the outside). This time, the drying time was estimated at 10 min. In case of blades, the temperature of the cylinders varied between 113 °C and 118 °C, while the drying temperature ranged from 100 °C to 102 °C, the average difference being of 15.5 K. The consumed power for driving the mobile cylinder with blades was experimentally measured at no more than 6 W (the starting and the continuous driving effort were included as well).

In order to compare the two constructive variants of tester (without and with blades), there were first calculated the useful heat necessary for wsd drying, $Q_{u}=19\;\mathrm{kcal}≅79,530\;J$, and the heating surface, $S=0,0377\;m^{2}$. Then, the global heat transfer coefficient was estimated by using the thermal transfer relationship:

(44) $$K=\frac{Q_{u}}{S\cdot \Delta T\cdot \tau }$$

where ΔT∈{46,15.5} K is the temperature difference in the observation range, and τ∈{19,10} min is the observation duration. Replacing the notation of this coefficient with $K_{b}^{-}$ for the case without blades, respectively $K_{b}^{+}$ for the case with blades, one obtains:

(45) $$\begin{array}{c} K_{b}^{-}=\frac{79,530}{0,0377\times 46\times 19\times 60}\;\frac{W}{m^{2}K}≅40.23\;\frac{W}{m^{2}K};\\ K_{b}^{+}=\frac{79,530}{0,0377\times 15.5\times 10\times 60}\;\frac{W}{m^{2}K}≅226.83\;\frac{W}{m^{2}K}. \end{array}$$

Consequently, $K_{b}^{+}$ is about 5.64 times greater than $K_{b}^{-}$. This proves that the use of blades leads to a more efficient solution. Thanks to heat transfer intensity increasing, the volume of GR was significantly reduced by 564%, which is a remarkable outcome. The experimental GR, constructed at a different scale from the tester, confirmed the results obtained above with the tester. Some of these special blades contribute to the wsd pushing and are arranged on 1-2 rows covering the entire active height, estimated at maximum 1 m. Within the GR, the processing temperature of wsd is about 1000°C and the heat transfer intensification, predicted by the tester, was confirmed during the experiments. 

### 2.5. Operating HYRON

Before starting the HYRON installation, some technological operations required for the plant functioning, should be executed for some components. The most important one is the coppering of absorbent liquid path, following the procedure below:

1. Fill A and D with grain filling, taking care to reach 50 cm high. Close the caps of A and D.
2. Prepare 925 kg aqueous copper sulphate solution (CuSO_4_ + 5H_2_O) of about 2% concentration (i.e., 25 kg of salt with 900 kg clean water) in a plastic bowl.
3. Provide the modular block A-HE-D with aqueous copper sulphate solution, ensuring that all valves are open, but the valve between the compressor C_2_ and A, which is kept closed. Close the latter valve, in order not to flood the buffer tank of C_2_ with absorbent liquid, which would automatically lead to the loss of fluid from its established path.
4. Connect RP to the electric power and start it. Recirculate the solution for 60 min throughout the whole block, during which a redox chemical reaction occurs, for depositing a 20 μm copper layer on all the internal surfaces of the assembly. Meantime, check:
  - if RP operates in different working regimes by adjusting the controller and the purging valve;
  - if the modular assembly is sealed and does not have leaks.
5. Evacuate the solution and store it separately for other possible and subsequent coppering.
6. Rinse the A-HE-D assembly with 900 l of drinking water, for 15–30 min.
7. Prepare 815.5 kg of copper-ammonium solution (according to the chemical recipe in Table 2) and fill in the modular assembly. Recirculate the solution.

The total power is of 70 kW, while the installed power is of 75 kW. One has to outline that the grid supplies only 5 kW of this power. In industry, heat recovery can be achieved from its own plant, for example for raw material preheating. In this way, electrical power can be reduced significantly. Various fuel generators can be used, as well.

After the technological tests have been carried out for each component of the plant (these are all preliminary actions), the technological process is started following the procedure below:

1. Start the modular assembly A-HE-D and RP for the recirculation of the absorbent liquid, as it has to reach a certain thermal regime in the desorber (55–60 °C). Prepare it before the syngas H_2_ + CO + CO_2_ + H_2_O^vapors^ is generated, to be able to intake, separate and distribute the hydrogen towards storing and IR, as well as the mix CO+CO_2_ to the furnace. Close the exhausting valves, the liquid supply valve, and the valve between the compressor C_2_ and A.
2. Start the compressors C_1_–C_3_ and adjust them to the established technological regime conditions. Simultaneously, open the cooling water valves of C and GRHEW.
3. Ignite F by using a fuel gas cylinder, to initiate the thermal regime and heat the fixed module of GR, as well as the iron ore pre-heater. When the provided sawdust reaches the GR surfaces, they have to be hot. After step 6, the furnace operates independently, being supplied by D (no more gas cylinder is needed).
4. Start the mechanical drive of GR and feed GR with raw material (wsd).
5. Start the mechanical drives of the mobile sieves in IR and feed IR with iron ore (hematite). Simultaneously, start the electrical resistances of IR and D, to provide the required heat intake in the reaction zone between ore and hydrogen.
6. Open the valve between C_2_ and A (that was closed) and adjust the DP_1,2_.
7. Start VP and the valve between the s-Fe-p collecting zone from IR and RRP.
8. When RRP is full, close the upper valve and VP, then flood the room with Ar. After 10 min, empty the room full of s-Fe-p by packing the product in voided bags and then restart VP, by opening the corresponding valve.
9. During the nominal regime functioning, periodically check the purge of C, to verify if it is correctly evacuating the condensate or not. Verify the evolution of the mud exhaust from GRHEW.

## 3. Results and Discussion

### 3.1. Experimental Setting

**Figure 3:**
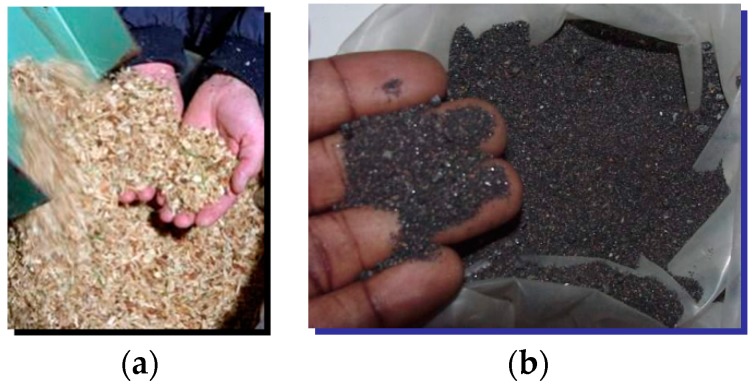
Wet sawdust with 35% humidity (**left**) and hematite with 180 μm granularity (**right**).

In nominal operating mode, the HYRON plant is able to produce up to 15 m^3^_n_/h h-p-H_2_ and up to 20 kg/h s-Fe-p, both of high purity (over 95% for hydrogen and over 88% for iron powder). The s-Fe-p has the shape of a metallic thick felt. The products are obtained from two main raw materials: sawdust with humidity of about 35% (wsd) and shredded iron ore (hematite) with an average granulation varying between 20 μm and 200 μm. These raw materials are illustrated in Figure 3.

The wsd characteristics are quite usual. For example, in [53], the results of proximate and ultimate analysis on sawdust are synthesized in page 169. They correspond to the characteristics of wsd employed to perform experiments with HYRON. The ultimate analysis clearly exhibits that the participations of other elements than carbon, hydrogen and oxygen are negligible. Other publications (such as [54] or [55]) lead to similar conclusions.

Both raw materials are available on the market at acceptable prices. For example, one can find: sawdust at average price of 20 €/t and hematite at average price of 180 €/t [56] or even of 75 $/t [57].

As technological parameters, one can say that GR consumes approximately 19 kg/h wsd (with 35% humidity) and, in nominal functioning, produces 28.7 m^3^_n_/h syngas.

In order to obtain the prescribed production of h-p-H2 and s-Fe-p, it is necessary to rhythmically feed the GR and IR bunkers with the raw material, according to Table 3. 

Initially, all components are at ambient temperature. For each of GR and IR components, a well-homogenized mixture of sawdust and, respectively, ore is prepared, by testing its corresponding characteristics. Simultaneously, one ensures the bunker filling and the stock to perform continuous functioning for up to 6–8 h, with recharge before any process resuming. The heating resistors of each of the two blocks are powered, in order to achieve the corresponding nominal temperature. The flow of sawdust and ore is initiated at GR and IR, respectively. After approximately 3 h, on the first start of the plant, the flow of s-Fe-p from IR is initiated as well. Subsequently, the durations are adjusted accordingly. Obviously, in order to produce s-Fe-p in permanent functioning mode, GR and IR have to work simultaneously. 

### 3.2. Analysis of Final Products

After obtaining the first batches of h-p-H_2_ and s-Fe-p, the results obtained with the tester were mostly confirmed. Two samples of h-p-H_2_ (gas in closed tubes) and s-Fe-p (as metallic thick felt), were collected and sent to some specialized laboratories that analyzed the composition. The first pair of samples was gathered before performing any adjustments on HYRON installation (Prototype, Hydrotech S.A., Bucharest, Romania), other than those resulting from the theoretical design of the technology. At the second pair, the components of HYRON plant were calibrated better. The results obtained from these pairs of samples are summarized in Table 4. 

Following recalibration, the purity of both products increased by approximately 2.5–3.5%. Hydrogen purity is quite high. For the iron, the technological process can be improved to achieve purity close to 93–95%. Note that there are several factors that could contribute to decreasing s-Fe-p purity. One of them is the absorbent liquid, which, in these experiments, was chosen less efficiently, due to a trade-off, because of the high price of reagents (especially of cuprous chloride). Other factor is the fine tuning of installation components, which can be improved. However, one expects that, in the absence of automatic control, s-Fe-p purity will not improve significantly. One of the important issues to solve is concerned with the design of a plant automatic control system. 

Finally, a technical-economic analysis of the process should be investigated as well. One can estimate the price of 1000 t h-p-H_2_ ($P_{{h-p-H}_{2}}$) and the price of 1 t s-Fe-p ($P_{s-\mathrm{Fe}-p}$) produced by using the proposed technology. In this respect, some preliminary data should be specified. The estimated power consumptions of HYRON installation are: $E_{{h-p-H}_{2}}≅3.34\;\mathrm{Gcal}≅14\;\mathrm{GJ}$ for 1000 m^3^_n_ h-p-H_2_ and $E_{s-\mathrm{Fe}-p}≅2\;\mathrm{Gcal}≅8.4\;\mathrm{GJ}$ for 1 t s-Fe-p, the efficiency being of η≅90%. Assume that the following prices are known: $P_{\mathrm{wsd}}$ [€/t] for wsd, $P_{\mathrm{ir}}$ (€/t) for iron ore and $P_{E}$ (€/Gcal) for energy. Moreover, recall that, within 1 h, the HYRON installation is able to produce 15 m^3^_n_ h-p-H_2_ and 20 kg s-Fe-p, by using 19 kg/h wsd (with 35% humidity) and 29 kg/h hematite. From the 15 m^3^_n_ h-p-H_2_, about 12 m^3^_n_ h-p-H_2_ are sent to the IR, in order to contribute to the s-Fe-p production. Then the prices $P_{\mathrm{ir}}$ and $P_{E}$ can be estimated as follows:

(46) $$\left\{ \begin{array}{l} P_{{h-p-H}_{2}}=\alpha \;P_{\mathrm{wsd}}+\frac{E_{{h-p-H}_{2}}}{\eta }P_{E}\\ P_{s-\mathrm{Fe}-p}=\beta \;P_{\mathrm{ir}}+\gamma \;P_{{h-p-H}_{2}}+\frac{E_{s-\mathrm{Fe}-p}}{\eta }P_{E} \end{array}\right.$$

where: α=0.019/0.015/0.65≅1.95 t/1000 m^3^_n_, β=29/20=1.45 and γ=0.012/0.02=0.6. 

Suppose that $P_{\mathrm{wsd}}=20$ €/t, $P_{\mathrm{ir}}=180$ €/t and $P_{E}=25$ €/Gcal. From Equations (46), one obtains: $P_{{h-p-H}_{2}}≅132$ €/1000 m^3^_n_ h-p-H_2_ and $P_{s-\mathrm{Fe}-p}≅456$ €/t s-Fe-p. This allows selling the HYRON products with affordable prices on the market. 

## 4. Conclusions and Perspectives

In this article, an innovative, cost-effective technology for simultaneous production of two substances with many practical applications was presented: high purity hydrogen (as a clean energy source) and soft iron powder (as a raw material for other final products). The experimental results show that both obtained substances are highly pure. The proposed technology emerges as a stand-alone achievement that can be valued both in education, scientific research and market. The HYRON installation described in the article, which allows implementation of the proposed technology, demonstrates its feasibility. 

Some perspectives for continuing research and exploiting its results are considered. Firstly, increasing the efficiency of HYRON plant can be accomplished through automation, even if it leads to a growth of the initial investment in the technology implementation. Automation can be realized by providing flow, level, pressure and temperature sensors, followed by the design and implementation of a microcontroller that optimally regulates these parameters. Secondly, one can aim to optimize the redox processes underlying the production of iron powder in order to increase its purity.

Although not all (actually quite many) HYRON design aspects were detailed in the article, there is a hope that specialists and practitioners in the field will be able to valorize the results of this research.

## 5. Patents 

There are two patents resulting from the work reported in this manuscript, as follows.

Stanasila, C.V.; Stanasila, O.N.; Stefanoiu, H.G.; Stefanoiu, D.; Munteanu, V. Method to Produce High Purity Hydrogen (Procedeu de obtinere a hidrogenului de inalta puritate), RO patent no. 127283, **2017**, WOS-DERWENT: 2012-G18484 (patent published in Romanian).

Stanasila, C.V.; Stanasila, O.N.; Stefanoiu, H.G.; Stefanoiu, D. Method and Installation to Reduce some Iron Ores Powders (Procedeu si instalatie de reducere a unor pulberi de minereu de fier), RO patent no. 128753, **2017**, WOS-DERWENT: 2013-N13013 (patent published in Romanian).

## Figures and Tables

**Table 1:** Experimental results of the apparent moving speed of the gas bubbles in the absorption filler.

$\mathrm{Column}\;\mathrm{Height}\;H_{c}\;(\mathrm{cm})$	$\mathrm{Gas}\;\mathrm{Flow}\;F_{g}(m^{3}/s)$	$\mathrm{Surface}\;S_{g}$ (m^2^)	$\mathrm{Apparent}\;\mathrm{Speed}\;v_{a}$ (m/s)
5	5.38 × 10^−4^	0.013^2^ × π	1.013
9	1.027 × 10^−3^	0.018^2^ × π	1.009
18	1.965 × 10^−3^	0.025^2^ × π	1.001
27	2.997 × 10^−3^	0.031^2^ × π	0.9927
36	3.788 × 10^−3^	0.035^2^ × π	0.9843
45	4.906 × 10^−3^	0.04^2^ × π	0.9760
50	5.383 × 10^−3^	0.042^2^ × π	0.9714

**Table 2:** Recipe of absorbent liquid.

Compound	Mass Flow (kg/h)	Number of Moles	Density (kg/m^3^)	Volume (m^3^)	Mass for Real Mixture (kg)
Ammonium (NH_3_, 24%)	190	2.6823+8.0222	910	0.1871	170.26
Cuprous chloride (CuCl)	167	1.6868	3530	0.0295	104.14
Ammonium chloride (NH_4_Cl)	209	3.9065	1527	0.0683	104.29
Clean water (H_2_O)	450	25	1000	0.4368	436.80
Total (copper-amonium mix)	1016	41.2978	–	0.7217	815.49

**Table 3:** Quantities of raw material to feed the HYRON installation during its normal functioning regime.

Raw Material	Main Characteristics	Quantity	Consumption Duration
Wet sawdust	35% (humidity)	19 kg	1 h
Hematite	20–200 µm (granularity)	29 kg	1 h
Copper-amonium mix	16.4% CuCl (concentration)	1 L	3 days

**Table 4:** Laboratory analysis results concerning the first two couples of samples (high purity hydrogen: h-p-H_2_, soft iron powder: s-Fe-p}.

Sample	H_2_ (%)	N (%)	Other Gases (%)	Fe (%)	Iron Oxides (%)	Other Compounds (%)
#1	97.174	2.260	0.566	85.3	14.628	0.072
#2	99.510	0.430	0.060	88.8	11.143	0.057

## Abbreviations

A	Absorber
A-HE-D	Absorber – Heat Exchanger - Desorber
BC	Buffer Cylinder
C	Cooler
D	Desorber
F	Furnace
GR	Gas Reactor
GRHEW	Gas Recycler with Heat Exchanger and Washer
HE	Heat Exchanger
HYRON	installation described within the article
IR	Iron Reactor
RP	Recycling Pump
RRP	Room to Reduce Pyrophoricity
VP	Void Pump
dsd	dry sawdust
h-p-H_2_	high purity hydrogen (H_2_)
s-Fe-p	soft iron (Fe) powder
syngas	synthesis gas
wsd	wet sawdust
